# Trends and Disparities in Mortality due to Diabetes Mellitus and Sepsis in the US Adults: 1999–2023

**DOI:** 10.1002/edm2.70082

**Published:** 2025-07-31

**Authors:** Asad Gul Rao, Sufyan Shahid, Neha Pervez, Ramsha Pervez, Raheel Ahmed

**Affiliations:** ^1^ Dow University of Health Sciences Karachi Pakistan; ^2^ Khawaja Muhammad Safdar Medical College Sialkot Pakistan; ^3^ Medical College Aga Khan University Karachi Pakistan; ^4^ National Heart and Lung Institute, Imperial College London London UK

**Keywords:** diabetes mellitus, disparities, epidemiology, mortality, sepsis, septic shock, United States

## Abstract

**Background:**

Diabetes mellitus (DM) increases susceptibility to infection and worsens outcomes in sepsis, a leading cause of preventable death. However, population‐level trends in sepsis‐related mortality among diabetic individuals in the United States (US) remain poorly characterised, especially in the context of the COVID‐19 pandemic. This study evaluates national patterns, temporal shifts, and demographic disparities in sepsis‐related mortality in diabetic patients from 1999 to 2023.

**Methods:**

We conducted a retrospective analysis using the Centers for Disease Control and Prevention Wide‐ranging Online Data for Epidemiologic Research (CDC WONDER) Multiple Cause of Death database. Sepsis‐related deaths with co‐listed DM were extracted for US adults between 1999 and 2023. Age‐adjusted mortality rates (AAMRs) were calculated and Joinpoint regression was used to estimate annual percentage changes (APCs) and identify significant trends.

**Results:**

A total of 483,207 sepsis‐related deaths occurred in individuals with DM during the study period. AAMRs declined significantly from 1999 to 2018 (APC: −1.22; *p* < 0.001), reversed sharply from 2018 to 2021 (APC: +18.14; *p* = 0.01), and declined again through 2023 (APC: −12.25; *p* < 0.001). Mortality was highest among older adults (AAMR: 32.63), males (9.72 vs. 7.80 in females), and non‐Hispanic Black and American Indian/Alaska Native populations (AAMRs: 17.94 and 17.92, respectively). Hispanic populations showed the steepest pandemic‐era increase (APC: +22.49) and subsequent decline (APC: −20.43). Rural areas consistently had higher AAMRs than urban areas (8.77 vs. 8.27), with sharper increases during the pandemic. State‐level disparities widened dramatically from 2021 to 2023, and regionally, the South and Midwest exhibited the highest and most persistent mortality burdens.

**Conclusion:**

Sepsis‐related mortality in diabetic individuals in the US has undergone dynamic shifts over the past 25 years, punctuated by COVID‐19 era surges and shaped by deep‐rooted demographic, geographic, and structural inequities. These findings warrant integrated diabetes‐infection care models, early sepsis recognition, and equity‐driven interventions to reduce mortality.

## Graphical abstract

Sepsis‐related mortality in US adults with diabetes declined from 1999 to 2018, surged sharply during the COVID‐19 pandemic, and fell again by 2023. Persistent disparities were observed by age, sex, race/ethnicity, and region.

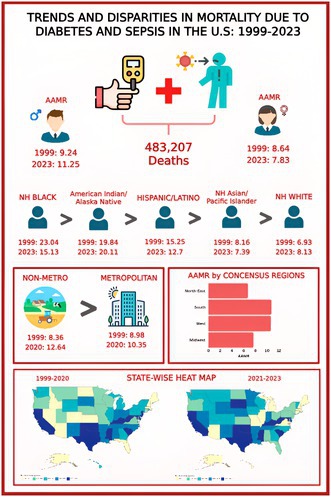

## 1 Introduction

Diabetes mellitus (DM) is a chronic metabolic disorder that affects multiple organ systems and impairs innate and adaptive immune function. In the United States (US) alone, over 38 million people—approximately 11.6% of the population—were living with diabetes as of 2023, with global prevalence projected to reach 643 million by 2030 [1, 2]. Persistent hyperglycaemia in diabetic individuals leads to immunosuppression through disrupted neutrophil function, cytokine signalling, and endothelial function [3].

Sepsis, a dysregulated host response to infection that can lead to multi‐organ failure and death, is one of the most serious complications in this population [4]. Glycaemic variability in diabetic individuals not only increases susceptibility to infection but is also an independent predictor of sepsis severity and mortality [5]. Despite advancements in critical care, sepsis remains a leading cause of mortality, accounting for over 200,000 deaths in the US in 2021 alone [6]. Diabetic patients experience disproportionately poor outcomes, accounting for an estimated 25% of all sepsis hospitalisations, with in‐hospital mortality rates exceeding 30% [7].

However, the long‐term epidemiological trends and demographic differences linked to DM‐related sepsis mortality have not been sufficiently recorded by national surveillance systems in the US. This intersection has become even more complex with the emergence of COVID‐19, since DM has been found to be a significant comorbidity in patients with acute respiratory distress syndrome (ARDS), viral sepsis, and increased intensive care unit (ICU) admission rates [8]. These challenges are further exacerbated by systemic disparities in healthcare access, delayed diagnosis, and the under‐recognition of early sepsis in diabetic patients [9].

Given the rising prevalence of DM and the persistent burden of sepsis, understanding their overlap is critical for informing clinical practice and public health policy. This study aims to assess national trends and demographic disparities and identify at‐risk populations and temporal shifts in sepsis‐related mortality among diabetic individuals in the US from 1999 to 2023.

## 2 Methods

### 2.1 Study Design and Population

This study examined national mortality trends involving both sepsis and DM in the US over a 25‐year period from 1999 to 2023. Mortality data were obtained from the National Vital Statistics System (NVSS) Multiple Cause of Death files, accessed via the CDC Wide‐Ranging Online Data for Epidemiologic Research (CDC WONDER) [10], which compiles non‐identifiable death certificate records from all 50 states and the District of Columbia. This database is widely used in epidemiological studies for its standardised and comprehensive reporting of mortality statistics across diverse population groups. The analysis included individuals aged 25 years and older whose death certificates listed both sepsis and DM as either the underlying or contributing causes of death. Through the Multiple Cause of Death Public Use Record, deaths were identified through the International Classification of Diseases, Tenth Revision (ICD‐10), with codes A40–A41 representing sepsis and E10–E14 representing DM. These ICD‐10 codes (A40–A41 for sepsis and E10–E14 for DM) have been consistently used in epidemiologic research to identify relevant deaths in administrative and mortality databases [11, 12]. Additionally, we adhered to the guidelines set forth by the Strengthening the Reporting of Observational Studies in Epidemiology (STROBE) reporting criteria [13]. As the dataset is publicly available and anonymised, the study was exempt from institutional review board (IRB) review.

### 2.2 Data Extraction

The dataset comprised annual population counts and demographic characteristics including sex, age, race and ethnicity, geographic region, level of urbanisation, and place of death. Racial and ethnic categories followed the CDC WONDER classification scheme and included Hispanic or Latino, non‐Hispanic (NH) White, NH Black or African American, and a combined group of other NH populations, such as Asian or Pacific Islander, Native Hawaiian, American Indian, or Alaska Native. Age was grouped into three brackets: 25–44 years (young adults), 45–64 years (middle‐aged adults), and 65 years and older (older adults). Geographic location was determined using US Census Bureau regional divisions, which categorise the US into the Northeast, Midwest, South, and West [14]. Urban–rural status was determined using the National Center for Health Statistics Urban–Rural Classification Scheme, which, based on the 2013 US Census definitions, categorised urban areas as large or medium/small metropolitan counties and rural areas as counties with populations under 50,000 [15]. Place of death was recorded in the dataset and categorised into hospitals, nursing facilities, private homes, hospice centres, or other/unspecified settings. All available data were incorporated into the analysis without any omissions, exclusions, or data suppression.

### 2.3 Statistical Analysis

To assess mortality trends linked to sepsis and DM, both crude mortality rates (CMRs) and age‐adjusted mortality rates (AAMRs) were calculated per 100,000 population annually from 1999 to 2023. CMRs were derived by dividing the total number of relevant deaths by the US population for each year. AAMRs were calculated using the 2000 US standard population for age adjustment, and 95% confidence intervals were reported for all estimates [16]. These metrics enabled comparison across different demographic subgroups and over time. Temporal changes in AAMRs were evaluated using Joinpoint Regression Program (Version 5.1.0, National Cancer Institute), which identifies inflection points and calculates annual percentage change (APC) with corresponding 95% confidence intervals [17]. A two‐tailed *p* < 0.05 was considered statistically significant. Log‐linear regression models were applied to assess the direction and significance of trends, with slopes differing from zero indicating meaningful increases or decreases.

## 3 Results

Between 1999 and 2023, there were 483,207 sepsis‐related deaths in diabetic patients (Table S1). The place of death was recorded for 476,334 cases: 81.33% occurred in medical facilities, 9.83% in nursing homes or long‐term care facilities, 5.25% in decedents' homes, and 3.59% in hospices (Table S2).

### 3.1 Demographic Trends in Mortality (Overall)

**FIGURE 1:**
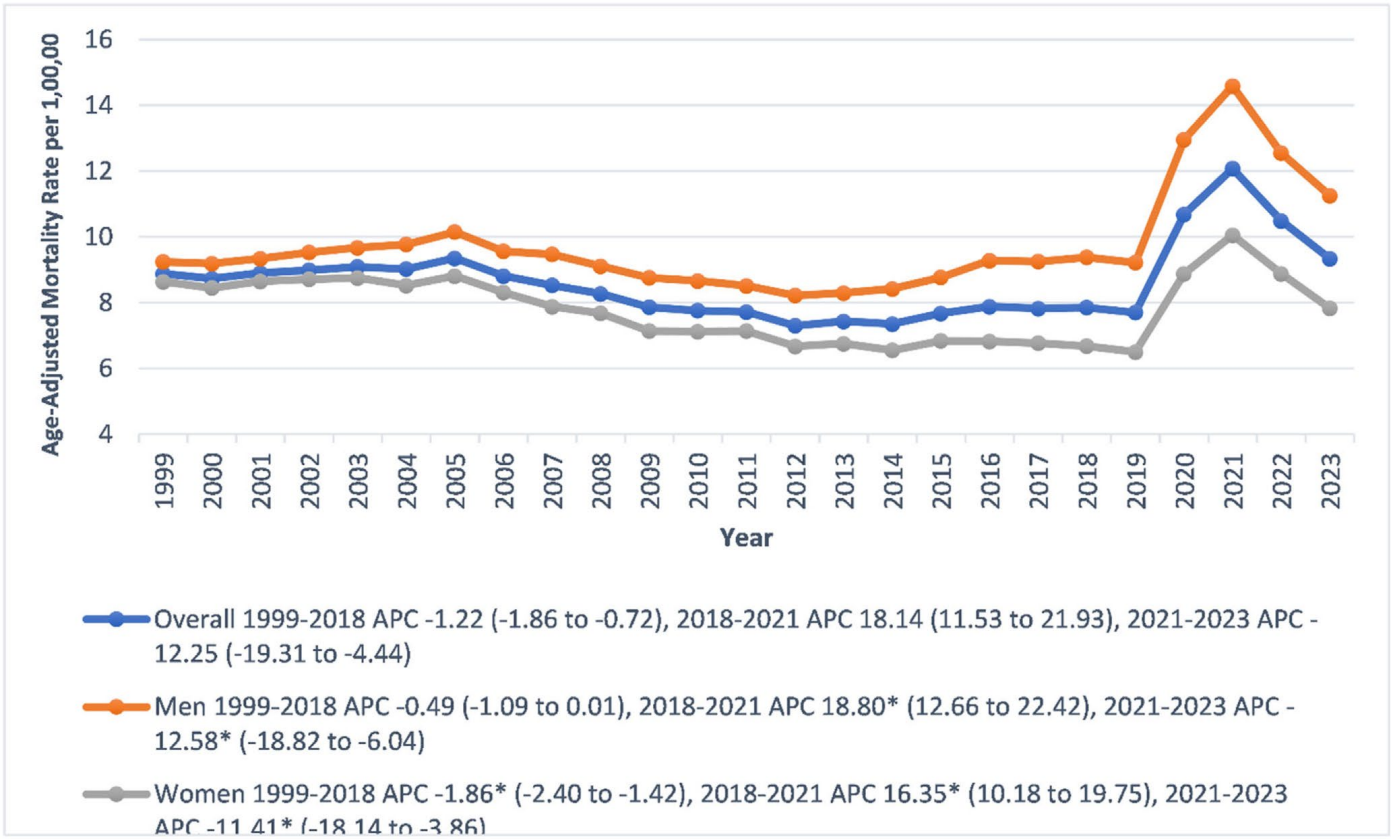
Overall and sex‐stratified age‐adjusted mortality rates per 100,000 for sepsis in diabetic patients in the United States, 1999 to 2023. * Indicates that the annual percentage change (APC) is significantly different from zero at *α* = 0.05. AAMR, age‐adjusted mortality rate.

The overall sepsis‐related AAMR in diabetic patients was 8.8 per 100,000 in 1999 and 9.3 in 2023. AAMR declined significantly from 1999 to 2018 (APC: −1.22; 95% CI: −1.86 to −0.72; *p* < 0.001), increased sharply during 2018–2021 (APC: 18.14; 95% CI: 11.53 to 21.93; *p* = 0.01), and declined again from 2021 to 2023 (APC: −12.25; 95% CI: −19.31 to −4.44; *p* < 0.001) (Figure 1, Tables S3 and S4).

### 3.2 Gender Stratification

**FIGURE 2:**
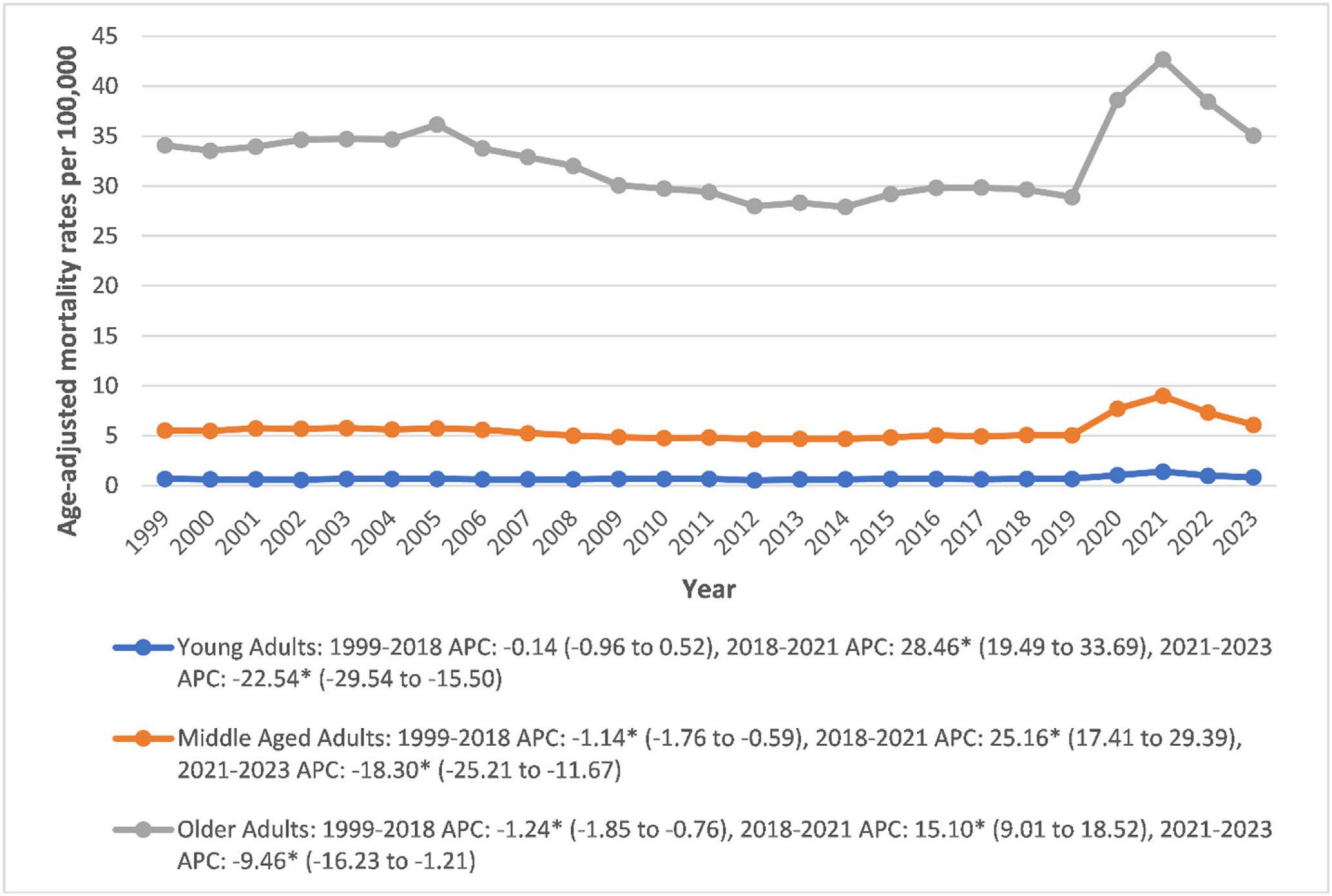
Age‐adjusted mortality rates per 100,000 for sepsis in diabetic patients, stratified by age in the United States, 1999 to 2023. * Indicates that the annual percentage change (APC) is significantly different from zero at *α* = 0.05. AAMR, age‐adjusted mortality rate.

Men consistently had higher AAMRs than women (9.72 vs. 7.80). In men, AAMR insignificantly decreased from 1999 to 2018 (APC: −0.49; 95% CI: −1.09 to 0.01; *p* = 0.7), rose significantly during 2018–2021 (APC: 18.80; 95% CI: 12.66 to 22.42; *p* = 0.001), and then declined (2021–2023: APC: −12.58; 95% CI: −18.82 to −6.04; *p* = 0.0001). In women, AAMR declined from 1999 to 2018 (APC: −1.86; 95% CI: −2.40 to −1.42; *p* = 0.001), then significantly increased from 2018 to 2021 (APC: 16.35; 95% CI: 10.18 to 19.75; *p* < 0.001), and then declined (2021–2023: APC: −11.41; 95% CI: −18.14 to −3.86; *p* < 0.001) (Figure 2, Tables S3 and S4).

### 3.3 Stratification by Age Groups

Older adults had the highest AAMRs (32.63), followed by middle‐aged (5.56) and young adults (0.71). In young adults, AAMR was stable from 1999 to 2018 (APC: −0.14; 95% CI: −0.96 to 0.52; *p* > 0.05), spiked in 2018–2021 (APC: 28.46; 95% CI: 19.49 to 33.69; *p* < 0.001), then declined (2021–2023: APC: −22.54; 95% CI: −29.54 to −15.50; *p* < 0.001). Middle‐aged adults saw a 1999–2018 decline (APC: −1.14; 95% CI: −1.76 to −0.59; *p* < 0.001), 2018–2021 increase (APC: 25.16; 95% CI: 17.41 to 29.39; *p* < 0.001), and 2021–2023 decline (APC: −18.30; 95% CI: −25.21 to −11.67; *p* < 0.001). Older adults followed a similar pattern, with an initial decline (1999–2018 APC: −1.24; 95% CI: −1.85 to −0.76; *p* < 0.001), a rise (2018–2021: APC: 15.10; 95% CI: 9.01 to 18.52; *p* < 0.001), and a subsequent drop (2021–2023: APC: −9.46; 95% CI: −16.23 to −1.21; *p* < 0.001) (Figure 2, Tables S3 and S5).

### 3.4 Racial Stratification

**FIGURE 3:**
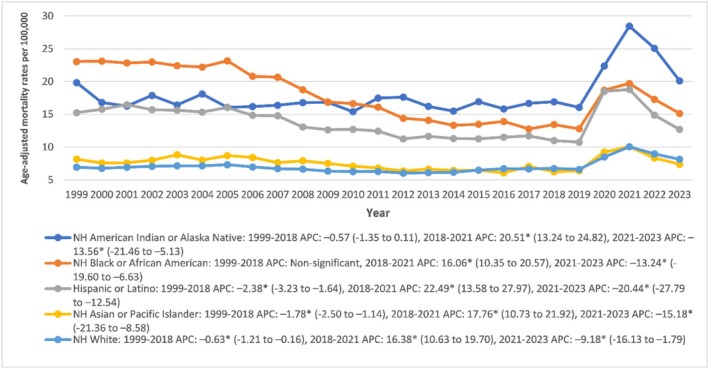
Age‐adjusted mortality rates per 100,000 for sepsis in diabetic patients, stratified by race in the United States, 1999 to 2023. * Indicates that the annual percentage change (APC) is significantly different from zero at *α* = 0.05. AAMR, age‐adjusted mortality rate.

Age‐adjusted mortality rates (AAMRs) were highest among non‐Hispanic (NH) Black individuals (17.94), followed closely by NH American Indian/Alaska Native populations (17.92), Hispanic individuals (13.84), NH Asian/Pacific Islanders (7.55), and NH Whites (7.02). Among NH American Indian/Alaska Native individuals, mortality rates showed no significant change from 1999 to 2018 (APC: −0.57; *p* > 0.05), followed by a marked increase from 2018 to 2021 (APC: 20.51; *p* < 0.001), and a significant decline from 2021 to 2023 (APC: −13.56; *p* < 0.001). NH Asian/Pacific Islanders exhibited a significant decline from 1999 to 2018 (APC: −1.78; *p* < 0.001), a sharp increase from 2018 to 2021 (APC: 17.76; *p* < 0.001), and another decline from 2021 to 2023 (APC: −15.18; *p* < 0.001). NH Black individuals experienced no significant change between 1999 and 2018 (*p* > 0.05), followed by a significant rise during 2018–2021 (APC: 16.06; *p* < 0.001), and a subsequent drop from 2021 to 2023 (APC: −13.24; *p* < 0.001). NH White populations showed a modest but significant decline from 1999 to 2018 (APC: −0.63; *p* < 0.001), a rise between 2018 and 2021 (APC: 16.38; *p* < 0.001), and a decrease in the 2021–2023 period (APC: −9.18; *p* < 0.001). Hispanic individuals experienced the most pronounced shifts, with a significant decline from 1999 to 2018 (APC: −2.38; *p* < 0.001), the steepest increase from 2018 to 2021 among all groups (APC: 22.49; *p* < 0.001), and a dramatic decline from 2021 to 2023 (APC: −20.44; *p* < 0.001) (Figure 3, Tables S3 and S6).

### 3.5 State‐Wise Distribution

From 1999 to 2020, age‐adjusted mortality rates (AAMRs) showed stark geographic disparities. States in the bottom 10% (Massachusetts, New Hampshire, Idaho, Florida, Alaska, and Maine) had rates of 4.91–5.44, while the top 90th percentile states (West Virginia, Mississippi, Texas, Kentucky, Oklahoma, and the District of Columbia) reached 11.25–18.78—a 2–4 fold difference.

During the 2021–2023 period, this geographic disparity intensified dramatically. The lowest‐rate states (New Hampshire, Alaska, Hawaii, Massachusetts, Connecticut, and Maine) now ranged from 2.08 to 6.22, contrasted with 15.92 to 28.27 in the new high‐burden group (South Dakota, District of Columbia, South Carolina, Mississippi, Kentucky, and Oklahoma). This represented a 3–14‐fold disparity, with Oklahoma's peak rate (28.27) exceeding Maine's lowest rate (2.08) by thirteen‐fold. While some high‐burden states like Mississippi and Kentucky remained problematic, new hotspots emerged in South Dakota and South Carolina during this later period (Figure S2, Table S7A).

### 3.6 Census Region

**FIGURE 4:**
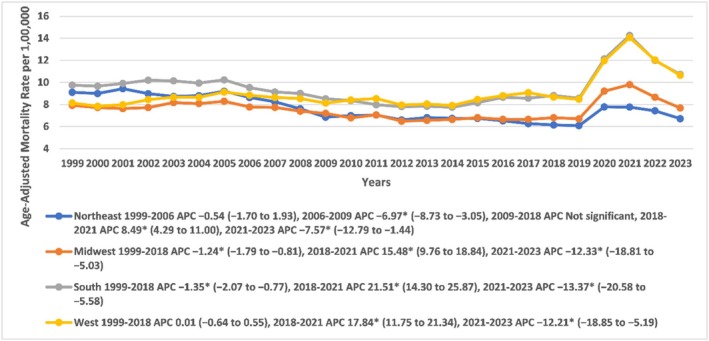
Age‐adjusted mortality rates per 100,000 for sepsis in diabetic patients, stratified by census regions in the United States, 1999 to 2023. * Indicates that the annual percentage change (APC) is significantly different from zero at *α* = 0.05. AAMR, age‐adjusted mortality rate.

Age‐adjusted mortality rates (AAMRs) showed distinct regional trends across US census regions. In the Northeast, rates were stable from 1999 to 2006 (APC: −0.5392, 95% CI: −1.7020 to 1.9339; *p* > 0.05), declined sharply through 2009 (APC: −6.9672, 95% CI: −8.7318 to −3.0496; *p* < 0.001), remained steady until 2018, then rose from 2018 to 2021 (APC: 8.4899, 95% CI: 4.2924 to 11.0014; *p* < 0.001) before declining again (APC: −7.5724, 95% CI: −12.7859 to −1.4382; *p* < 0.001). The Midwest, South, and West all experienced stable or declining trends through 2018, followed by sharp increases during 2018–2021 and declines from 2021 to 2023. While the Midwest and South had steady declines before 2018, the West showed no significant change until the pandemic‐era spike. These patterns reflect a shared disruption during COVID‐19 across regions despite prior variation (Figure 4, Tables S3 and S8).

### 3.7 Urbanisation

**FIGURE 5:**
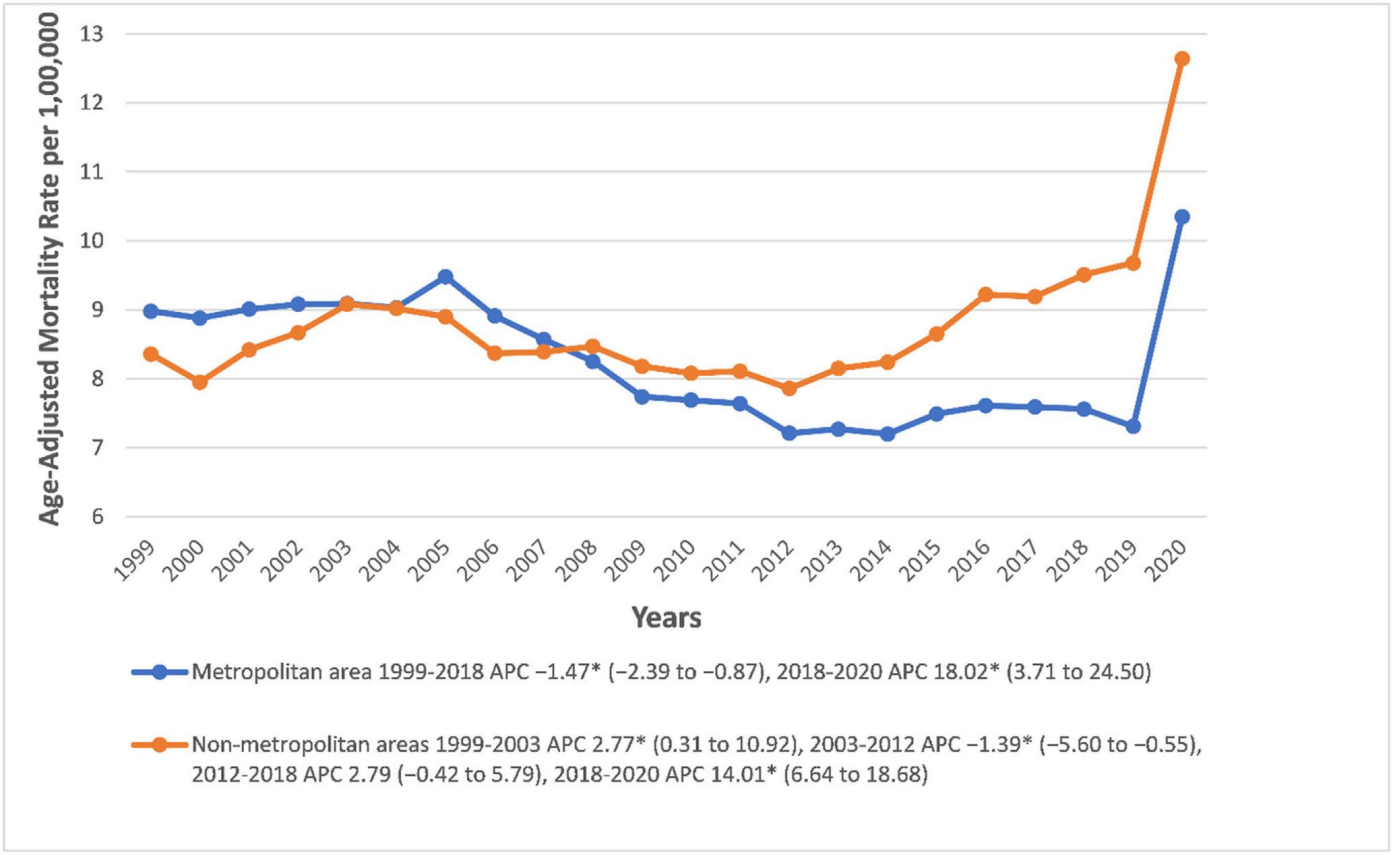
Sepsis‐related Age‐Adjusted Mortality Rates per 100,000 in Diabetic Patients in the Metropolitan and Non‐metropolitan areas in the United States, 1999 to 2020. * Indicates that the APC is significantly different from zero at *α* = 0.05. AAMR, age‐adjusted mortality rate.

Urban–rural mortality disparities were analysed for 1999–2020 due to unavailable data beyond 2020. Throughout this period, non‐metropolitan areas consistently bore higher mortality burdens than metropolitan areas (8.77 vs. 8.27). In metropolitan areas, AAMRs declined significantly from 1999 to 2018 (APC: −1.4724, 95% CI: −2.3883 to −0.8681; *p* < 0.001), then rose sharply during 2018–2020 (APC: 18.0217, 95% CI: 3.7060 to 24.4980; *p* < 0.001). Non‐metropolitan areas showed a more complex trajectory: an initial rise (1999–2003: APC: 2.7690, 95% CI: 0.3107 to 10.9189; *p* = 0.028), a significant decline (2003–2012: APC: −1.3862, 95% CI: −5.5976 to −0.5514; *p* = 0.002), a non‐significant increase (2012–2018), and a sharp rise from 2018 to 2020 (APC: 14.0050, 95% CI: 6.6382 to 18.6776; *p* < 0.001) (Figure 5, Tables S3 and S9).

## 4 Discussion

In this nationwide analysis of sepsis‐related mortality among individuals with DM from 1999 to 2023 in the US, we identified important trends, significant demographic and regional disparities, and a sharp inflection coinciding with the COVID‐19 pandemic. Our results suggested that the early 21st century was characterised by a steady but significant decline in AAMRs. However, this trend was abruptly reversed around 2018, and a substantial rise occurred through 2021 with a subsequent decline in 2023. This illustrates the intricate relationships among infectious disease dynamics, chronic disease management, the competence of the healthcare system, and the innate biological susceptibility of diabetic patients to sepsis.

From 1999 to 2018, the overall decline in sepsis‐related mortality among diabetic patients likely reflects improvements in DM management, expanded access to primary care, and growing adoption of standardised sepsis care protocols [18, 19, 20]. Advances in glycaemic control, particularly following the Food and Drug Administration's (FDA) approval of metformin in 1994, have contributed significantly to better DM management [21]. Additionally, updated guidelines such as Sepsis‐3 and the Surviving Sepsis Campaign (SSC) have improved sepsis awareness and quality of care, enabling enhanced recognition of sepsis and earlier initiation of antibiotics and fluid resuscitation, especially in vulnerable populations [22, 23, 24]. Broader healthcare reforms, including the Patient Protection and Affordable Care Act (ACA), further expanded access to health insurance in the US; an estimated 20 million previously uninsured individuals gained coverage [25]. However, despite these gains, individuals with DM remain uniquely susceptible to both the onset and severity of sepsis [26, 27].

This biological susceptibility may help explain why the 2018–2021 surge in mortality occurred. The COVID‐19 pandemic caused severe healthcare disruptions and led to delayed routine DM care, faltered preventive services, and medicine adherence [28, 29]. Meanwhile, SARS‐CoV‐2 infection itself acts as a strong immune and endothelial function disruptor, further destabilising diabetic patients already at high risk for dysregulated immune responses [30]. Collectively, in diabetic individuals who are already in a pro‐inflammatory and hypercoagulable state, such disruptions could lead to higher rates of septic complications, multi‐organ failure, and ultimately death [31]. Additionally, hospitals were overburdened by the pandemic, which overwhelmed ICUs and emergency departments, resulting in a shortage of critical care capacity [28, 32, 33]. The observed mortality decline from 2021 to 2023 may reflect the gradual restoration of healthcare infrastructure, resumption of outpatient diabetes management, and notably, widespread COVID‐19 vaccination rollout, which helped reduce severe infections, hospital burden, and associated septic deaths [34]. These system‐level recoveries likely enabled earlier infection detection, improved glycaemic control, and better critical care delivery—especially in vulnerable diabetic populations.

When stratified by sex, our findings consistently showed higher sepsis‐related mortality among men with DM compared to women. Biologically, men have demonstrated to have weaker innate and adaptive immune responses than women, with lower levels of oestrogen‐linked immunoprotection and higher levels of proinflammatory cytokine production [35, 36]. In the context of DM, these immunological deficits are exacerbated as men tend to have poorer glycaemic control, more visceral adiposity (driving systemic inflammation), and a higher prevalence of atherosclerosis, which can compromise perfusion and organ integrity during septic episodes [37, 38, 39]. Behaviourally, men are less likely to follow chronic illness management regimens or seek early medical attention, delaying diagnosis and treatment initiation [40, 41]. Additionally, evidence suggests that men are less likely to adopt preventive health practices or regular infection screenings, which increased their vulnerability during pandemic periods [41]. During the COVID‐19 era, these underlying susceptibilities may have been magnified, potentially explaining the steeper rise in male mortality rates during the pandemic and the comparatively smaller decline in male sepsis‐related deaths compared to females from 2021 to 2023.

Our results showed that older adults with DM had the highest sepsis‐related mortality burden, with AAMRs far exceeding those in younger populations. This pattern is expected given that aging independently impairs immune competence, vascular integrity, and organ reserve [42, 43, 44]. However, in the context of DM, these age‐related changes are often amplified. Older adults with DM are more likely to have advanced microvascular and macrovascular complications that can accelerate progression to septic shock and limit the body's ability to respond [45, 46]. Furthermore, many of the older adults live in long‐term care facilities, which faced high infection rates during the COVID‐19 pandemic, making them particularly vulnerable and exposed [47]. Although the overall mortality rate was lower for younger and middle‐aged adults, the significant increase in their AAMRs from 2018 to 2021 raises the possibility that pandemic‐related stressors, such as job loss, insulin inaccessibility, and healthcare avoidance, may have severely disrupted DM control, making them more vulnerable to infection and septic death [48, 49].

Our study demonstrated significant and persistent racial disparities in sepsis‐related deaths among DM patients, reflecting broader trends of health inequity in the US. NH Black and American Indian and Alaska Native populations experienced the highest AAMRs, while NH Asian and Pacific Islander and NH White populations had lower baseline AAMRs. Mortality trends in Hispanic or Latino populations were highly variable, with pre‐pandemic declines followed by the steepest increases during the pandemic among all groups. Systemic reasons such as greater prevalence of comorbid chronic illnesses, socioeconomic marginalisation, historical and current medical racism, limited access to primary and preventive care, and unstable housing are likely the cause of these discrepancies [50, 51, 52]. Additionally, studies suggest that Hispanic Americans and NH Black individuals are overrepresented in essential worker roles, which increased exposure risk during the COVID‐19 pandemic [53, 54]. Moreover, lower insurance coverage rates and language barriers may have delayed or complicated access to early sepsis care during pandemic surges. As per a report, Black and Latino individuals had the highest rates of uninsured status compared to White people, particularly after COVID‐19 [55].

The significant regional and state‐level variation in mortality rates found in our results emphasises the importance of social determinants, public health funding, and healthcare access in influencing sepsis outcomes in diabetic patients. Mississippi, Kentucky, Texas, Oklahoma, and South Carolina are among the states with consistently high AAMRs. These states also typically have greater baseline DM prevalence, higher rates of poverty and obesity, fewer healthcare providers per capita, and lower insurance coverage rates [56, 57, 58, 59, 60]. Many of these states have not adopted Medicaid expansion, which worsened inequities by restricting access to acute sepsis management therapies as well as preventative DM care [61]. The fact that South Dakota and South Carolina became new hotspots during the pandemic raises the possibility that these states' vulnerabilities were revealed by changes in population dynamics, the strain on the healthcare system, and perhaps pandemic policy responses (like postponed lockdowns, scaled‐back hospital capacity expansions, and limited public health messaging). At the broader regional levels, the Midwest and South were the most severely affected with the highest AAMRs, followed by the West and the Northeast with the least mortality. This is in line with the previous studies evaluating regional sepsis‐related mortality and revealed the highest rates in Southern regions [62, 63].

Our results also revealed that non‐metropolitan areas exhibited higher sepsis‐related mortality rates among diabetic individuals than metropolitan regions across the study period, a gap that widened considerably during the pandemic. Rural hospitals are often underfunded, understaffed, and poorly equipped for managing complex critical illness such as sepsis [64]. Similarly, patients with DM who live in remote locations may experience higher wait times at emergency departments, fewer chances for early infection screening, and restricted access to DM educators or endocrinologists. These system‐level issues are worsened by an increased prevalence of health‐risky habits like smoking and inactivity and socioeconomic obstacles, including insecure transportation and the digital divide that restricts telemedicine. Additionally, rural hospitals were more likely to face staffing and supply shortages and ICU overflow during the pandemic, which could have further delayed or decreased the quality of sepsis care [65, 66]. These geographic patterns also point toward a broader phenomenon that diabetic patients living in resource‐limited settings are more likely to suffer worse sepsis outcomes, regardless of personal risk profiles, due to systemic barriers to timely, high‐quality care.

Our study presents some valuable clinical and public health implications. First, clinicians must recognise that DM is an active biological booster of infection severity and sepsis progression, rather than just being a merely co‐morbid condition. The mechanisms at play highlight how crucial rigorous glycaemic management is for long‐term metabolic advantages as well as for infection resilience and survival. Second, the findings call for enhanced early recognition and management of infections in diabetic patients, particularly in older adults, men, and racial/ethnic minorities who display disproportionately high mortality rates. Regular use of sepsis screening technologies in emergency and inpatient settings, along with more stringent clinical routes that take patients' impaired immune systems into consideration, may improve time to antibiotics and slow the onset of septic shock. Third, the dramatic increase in mortality during COVID‐19 highlights the effects of fragmented treatment and delayed chronic illness management. Clinical systems must incorporate robust forms of DM care, such as telemedicine, mobile health units, and proactive patient outreach, especially in rural and underprivileged areas, to prevent similar outcomes during future healthcare disruptions. Fourth, our study highlights the importance of tailored care strategies for high‐risk subpopulations, including NH Black, Hispanic, and American Indian and Alaska Native patients, who face the greatest disparities in sepsis‐related death. Community‐based infection awareness programmes, language‐accessible education, and culturally competent care must be prioritised by clinicians and healthcare systems to improve engagement and outcomes in these populations. Finally, regional and rural disparities imply that where care is delivered is just as important as how it is delivered. Enhancing sepsis response capabilities in non‐metropolitan hospitals, such as access to critical care resources, standardised sepsis bundles, and faster transfer pathways to higher‐level centres, as well as better coordination between acute care systems and primary care providers, can help prevent avoidable sepsis escalation in diabetic patients and promote early infection detection.

## 5 Limitations

Our study has some limitations that should be considered. First, this is a retrospective study based on death certificate data from the CDC WONDER database, which may be subject to misclassification or underreporting. Specifically, although we used standardised ICD‐10 codes (E10–E14 for diabetes and A40–A41 for sepsis), changes in diagnostic criteria—such as the transition from Sepsis‐2 to Sepsis‐3—may have introduced temporal inconsistencies in coding practices, especially during transitional years. Second, the dataset does not include important clinical factors like body mass index, HbA1c levels, the existence of diabetic sequelae (such as neuropathy or nephropathy), or concurrent infections. These characteristics could have a significant impact on sepsis outcomes and susceptibility, but the analysis was unable to account for them. Third, data on urban versus rural residence (metropolitan vs. non‐metropolitan classification) were not available beyond 2020, restricting the ability to fully assess urban–rural disparities during the pandemic peak years (2021–2023). This gap may lead to underestimation or misinterpretation of geographic mortality trends during the COVID‐19 era. Lastly, death classification may have been affected by modifications to sepsis criteria (e.g., Sepsis‐2 vs. Sepsis‐3) or changes in ICD coding procedures during the study period, particularly during transition years. The observed patterns may be somewhat impacted by this temporal discrepancy.

## 6 Conclusion

This nationwide analysis of over two decades of mortality data reveals that sepsis‐related deaths among individuals with DM in the US remain a significant and evolving public health challenge. Although early advancements in sepsis treatment and DM management helped to steadily lower mortality, the COVID‐19 pandemic drastically reversed these gains and exposed the increased susceptibility of diabetic individuals to infectious threats. Significant disparities by age, sex, ethnicity, and geography are highlighted by our findings, demonstrating that outcomes are influenced by both structural inequity and biology. As DM continues to rise in prevalence and novel infections emerge, proactive strategies that integrate chronic disease management with sepsis prevention and health system resilience are essential.

## Author Contributions

Conceptualization: A.G.R.; data curation: A.G.R. and S.S.; formal analysis: S.S.; funding acquisition: N/A; investigation: A.G.R.; methodology: N.P. and A.G.R.; project administration: A.G.R.; resources: A.G.R, S.S. and N.P.; software: S.S.; supervision: R.A.; validation: R.A.; visualisation: A.G.R.; writing – original draft: A.G.R, S.S, N.P, R.P.; writing – review and editing: R.A, A.G.R, R.P.

## Conflicts of Interest

The authors declare no conflicts of interest.

## Supporting information


**Table S1:** Sepsis and Diabetes–related Mortality, Stratified by Sex and Race in Adults in the United States, 1999 to 2023.
**Table S2:** Sepsis and Diabetes–related Mortality, Stratified by Place of Death in Adults in the United States, 1999 to 2023.
**Table S3:** Annual percent change (APC) of Sepsis and Diabetes–related Age‐Adjusted Mortality Rates per 100,000 in Adults in the United States, 1999 to 2023.
**Table S4:** Overall and Sex‐Stratified Sepsis and Diabetes–related Age‐Adjusted Mortality Rates per 100,000 in Adults in the United States from 1999 to 2023.
**Table S5:** Sepsis and Diabetes–related Mortality, Stratified by Age group in Adults in the United States, 1999 to 2023.
**Table S6:** Sepsis and Diabetes–related Age‐Adjusted Mortality Rates per 100,000 stratified by Race in Adults in the United States from 1999 to 2023.
**Table S7A:** Sepsis and Diabetes–related Age‐Adjusted Mortality Rates per 100,000 Stratified by State in Adults in the United States, 1999 to 2020.
**Table S7B:** Sepsis and Diabetes–related Age‐Adjusted Mortality Rates per 100,000 Stratified by State in Adults in the United States, 2021 to 2023.
**Table S8:** Sepsis and Diabetes–related Age‐Adjusted Mortality Rate per 100,000 Stratified by Census Region in Adults in the United States 1999–2023.
**Table S9:** Overall Sepsis and Diabetes–related Age‐Adjusted Mortality Rates per 100,000 in Adults in the Metropolitan and Non‐metropolitan areas in the United States, 1999 to 2020.
**Figure S1:** Diabetes Mellitus and Sepsis‐Related Annual Percentage Change (APC) in the United States from 1999 to 2023 stratified by (A) Overall (B) Sex (C) Race (D) Urbanisation (E) Consensus Region (F) Age.
**Figure S2:** State‐wise distribution of sepsis related mortality in diabetic patients (A) 1999–2020 (B) 2021–2023.

## Data Availability

The data that support the findings of this study are openly available in CDC WONDER at https://wonder.cdc.gov/.
