# Supplementary material for: Trends and Disparities in Mortality due to Diabetes Mellitus and Sepsis in the US Adults: 1999–2023
Source: Endocrinol Diabetes Metab. 2025 Jul 31;8(5):e70082. doi: 10.1002/edm2.70082 (PMC12312802; doi:10.1002/edm2.70082)
Supplement: Supplementary file 1 — Table S1: Sepsis and Diabetes–related Mortality, Stratified by Sex and Race in Adults in the United States, 1999 to 2023. Table S2: Sepsis and Diabetes–related Mortality, Stratified by Place of Death in Adults in the United States, 1999 to 2023. Table S3: Annual percent change (APC) of Sepsis and Diabetes–related Age‐Adjusted Mortality Rates per 100,000 in Adults in the United States, 1999 to 2023. Table S4: Overall and Sex‐Stratified Sepsis and Diabetes–related Age‐Adjusted Mortality Rates per 100,000 in Adults in the United States from 1999 to 2023. Table S5: Sepsis and Diabetes–related Mortality, Stratified by Age group in Adults in the United States, 1999 to 2023. Table S6: Sepsis and Diabetes–related Age‐Adjusted Mortality Rates per 100,000 stratified by Race in Adults in the United States from 1999 to 2023. Table S7A: Sepsis and Diabetes–related Age‐Adjusted Mortality Rates per 100,000 Stratified by State in Adults in the United States, 1999 to 2020. Table S7B: Sepsis and Diabetes–related Age‐Adjusted Mortality Rates per 100,000 Stratified by State in Adults in the United States, 2021 to 2023. Table S8: Sepsis and Diabetes–related Age‐Adjusted Mortality Rate per 100,000 Stratified by Census Region in Adults in the United States 1999–2023. Table S9: Overall Sepsis and Diabetes–related Age‐Adjusted Mortality Rates per 100,000 in Adults in the Metropolitan and Non‐metropolitan areas in the United States, 1999 to 2020. Figure S1: Diabetes Mellitus and Sepsis‐Related Annual Percentage Change (APC) in the United States from 1999 to 2023 stratified by (A) Overall (B) Sex (C) Race (D) Urbanisation (E) Consensus Region (F) Age. Figure S2: State‐wise distribution of sepsis related mortality in diabetic patients (A) 1999–2020 (B) 2021–2023. [file EDM2-8-e70082-s001.docx]

**Supplementary Appendix**

**Supplemental Table 1** Sepsis and Diabetes–related Mortality, Stratified by Sex and Race in Adults in the United States, 1999 to 2023.

| **Deaths** | | | | | | | | | |
| --- | --- | --- | --- | --- | --- | --- | --- | --- | --- |
| **Year** | **Overall** | **Women** | **Men** | **NH White** | **NH Black or African American** | **NH Asian or Pacific Islander** | **NH American Indian or Alaska Native** | **Hispanic or Latino** | **Population** |
| 1999 | 15670 | 8958 | 6712 | 10235 | 3507 | 326 | 159 | 1397 | 180408769 |
| 2000 | 15601 | 8855 | 6746 | 10057 | 3534 | 326 | 142 | 1492 | 181984640 |
| 2001 | 16194 | 9153 | 7041 | 10416 | 3588 | 341 | 142 | 1656 | 184305128 |
| 2002 | 16679 | 9354 | 7325 | 10724 | 3663 | 400 | 163 | 1671 | 186208028 |
| 2003 | 17131 | 9513 | 7618 | 11031 | 3650 | 461 | 162 | 1786 | 188090429 |
| 2004 | 17248 | 9426 | 7822 | 11086 | 3678 | 439 | 182 | 1817 | 190205384 |
| 2005 | 18236 | 9879 | 8357 | 11539 | 3955 | 513 | 171 | 2013 | 192551384 |
| 2006 | 17511 | 9468 | 8043 | 11153 | 3661 | 525 | 175 | 1963 | 195019359 |
| 2007 | 17283 | 9136 | 8147 | 10882 | 3689 | 488 | 177 | 2020 | 197403777 |
| 2008 | 17082 | 9062 | 8020 | 10971 | 3445 | 534 | 181 | 1924 | 199795090 |
| 2009 | 16467 | 8562 | 7905 | 10570 | 3198 | 542 | 190 | 1927 | 202107016 |
| 2010 | 16591 | 8646 | 7945 | 10602 | 3208 | 536 | 190 | 2013 | 203891983 |
| 2011 | 16960 | 8863 | 8097 | 10807 | 3197 | 563 | 211 | 2139 | 206592936 |
| 2012 | 16509 | 8504 | 8005 | 10650 | 2983 | 552 | 229 | 2035 | 208826037 |
| 2013 | 17045 | 8738 | 8307 | 10926 | 3013 | 612 | 228 | 2208 | 211085314 |
| 2014 | 17375 | 8650 | 8725 | 11177 | 2973 | 633 | 221 | 2284 | 213809280 |
| 2015 | 18527 | 9251 | 9276 | 12007 | 3102 | 682 | 243 | 2405 | 216553817 |
| 2016 | 19390 | 9384 | 10006 | 12516 | 3301 | 679 | 244 | 2576 | 218641417 |
| 2017 | 19867 | 9557 | 10310 | 12792 | 3142 | 831 | 267 | 2744 | 221447331 |
| 2018 | 20217 | 9578 | 10639 | 13015 | 3401 | 784 | 280 | 2683 | 223311190 |
| 2019 | 20257 | 9513 | 10744 | 13036 | 3312 | 838 | 272 | 2754 | 224981167 |
| 2020 | 28663 | 13132 | 15531 | 16849 | 5018 | 1280 | 407 | 5056 | 226635013 |
| 2021 | 32015 | 14519 | 17496 | 19319 | 5242 | 1362 | 467 | 5365 | 228238412 |
| 2022 | 28745 | 13371 | 15374 | 17948 | 4658 | 1197 | 427 | 4255 | 229508599 |
| 2023 | 25944 | 11936 | 14008 | 16319 | 4171 | 1104 | 349 | 3767 | 231529762 |
| **Total** | 483207 | 245008 | 238199 | 306627 | 90289 | 16548 | 5879 | 61950 | 5163131262 |

NH, non-Hispanic.

**Supplemental Table 2.** Sepsis and Diabetes–related Mortality, Stratified by Place of Death in Adults in the United States, 1999 to 2023

| **Deaths** | | | | |
| --- | --- | --- | --- | --- |
| **Year** | **Medical**  **Facility** | **Nursing Home/Long-term**  **Care Facility** | **Hospices** | **Home** |
| 1999 | 12802 | 2242 | N/A | 502 |
| 2000 | 12800 | 2204 | N/A | 488 |
| 2001 | 13346 | 2176 | N/A | 520 |
| 2002 | 13948 | 2026 | N/A | 520 |
| 2003 | 14073 | 2192 | 10 | 577 |
| 2004 | 14266 | 2039 | 37 | 599 |
| 2005 | 14980 | 2197 | 78 | 684 |
| 2006 | 14500 | 1979 | 162 | 630 |
| 2007 | 14386 | 1819 | 225 | 634 |
| 2008 | 13878 | 1715 | 301 | 689 |
| 2009 | 13107 | 1708 | 359 | 688 |
| 2010 | 13573 | 1655 | 405 | 709 |
| 2011 | 13689 | 1654 | 548 | 806 |
| 2012 | 13140 | 1671 | 644 | 756 |
| 2013 | 13421 | 1732 | 693 | 887 |
| 2014 | 13689 | 1692 | 850 | 941 |
| 2015 | 14511 | 1793 | 1066 | 1005 |
| 2016 | 15009 | 1811 | 1187 | 1167 |
| 2017 | 15470 | 1786 | 1227 | 1162 |
| 2018 | 15651 | 1808 | 1236 | 1326 |
| 2019 | 15553 | 1758 | 1363 | 1350 |
| 2020 | 22662 | 2024 | 1516 | 2102 |
| 2021 | 25928 | 1771 | 1692 | 2218 |
| 2022 | 22805 | 1748 | 1703 | 2075 |
| 2023 | 20224 | 1645 | 1785 | 1956 |
| **Total** | 387411 | 46845 | 17087 | 24991 |

**Supplemental Table 3** Annual percent change (APC) of Sepsis and Diabetes–related Age-Adjusted Mortality Rates per 100,000 in Adults in the United States, 1999 to 2023

| **Year Interval** | **APC (95% CI)** | **P value** |
| --- | --- | --- |
| **Overall** | | |
| 1999-2018 | -1.22 (-1.86 to -0.72) | < 0.001 |
| 2018-2021 | 18.14 (11.53 to 21.93) | < 0.001 |
| 2021-2023 | -12.25 (-19.31 to -4.44) | < 0.001 |
| **Men** | | |
| 1999-2018 | -0.49 (-1.09 to 0.01) | p>0.05 |
| 2018-2021 | 18.80 (12.66 to 22.42) | < 0.001 |
| 2021-2023 | -12.58 (-18.82 to -6.04) | < 0.001 |
| **Women** | | |
| 1999-2018 | -1.86 (-2.40 to -1.42) | < 0.001 |
| 2018-2021 | 16.35 (10.18 to 19.75) | < 0.001 |
| 2021-2023 | -11.41 (-18.14 to -3.86) | < 0.001 |
| **Young Adults (25-44 years)** | | |
| 1999-2018 | -0.14 (-0.96 to 0.52) | p>0.05 |
| 2018-2021 | 28.46 (19.49 to 33.69) | < 0.001 |
| 2021-2023 | -22.54 (-29.54 to -15.50) | < 0.001 |
| **Middle Aged Adults (45-64 years)** | | |
| 1999-2018 | -1.14 (-1.76 to -0.59) | < 0.001 |
| 2018-2021 | 25.16 (17.41 to 29.39) | < 0.001 |
| 2021-2023 | -18.30 (-25.21 to -11.67) | < 0.001 |
| **Older Adults (65 years and above)** | | |
| 1999-2018 | -1.24 (-1.85 to -0.76) | < 0.001 |
| 2018-2021 | 15.10 (9.01 to 18.52) | < 0.001 |
| 2021-2023 | -9.46 (-16.23 to -1.21) | < 0.001 |
| **NH American Indian or Alaska Native** | | |
| 1999-2018 | −0.57 (−1.35 to 0.11) | p>0.05 |
| 2018-2021 | 20.51 (13.24 to 24.82) | < 0.001 |
| 2021-2023 | −13.56 (−21.46 to −5.13) | < 0.001 |
| **NH Black or African American** | | |
| 1999-2018 | Not Significant |  |
| 2018-2021 | 16.06 (10.35 to 20.57) | < 0.001 |
| 2021-2023 | −13.24 (−19.60 to −6.63) | < 0.001 |
| **Hispanic or Latino** | | |
| 1999-2018 | −2.38 (−3.23 to −1.64) | < 0.001 |
| 2018-2021 | 22.49 (13.58 to 27.97) | < 0.001 |
| 2021-2023 | −20.44 (−27.79 to −12.54) | < 0.001 |
| **NH Asian or Pacific Islander** | | |
| 1999-2018 | −1.78 (−2.50 to −1.14) | < 0.001 |
| 2018-2021 | 17.76 (10.73 to 21.92) | < 0.001 |
| 2021-2023 | −15.18 (−21.36 to −8.58) | < 0.001 |
| **NH White** | | |
| 1999-2018 | −0.63 (−1.21 to −0.16) | < 0.001 |
| 2018-2021 | 16.38 (10.63 to 19.70) | < 0.001 |
| 2021-2023 | −9.18 (−16.13 to −1.79) | < 0.001 |
| **Non-metropolitan areas** | | |
| 1999-2003 | 2.77 (0.31 to 10.92) | < 0.001 |
| 2003-2012 | −1.39 (−5.60 to −0.55) | < 0.001 |
| 2012-2018 | 2.79 (−0.42 to 5.79) | p>0.05 |
| 2018-2020 | 14.01 (6.64 to 18.68) | < 0.001 |
| **Metropolitan area** | | |
| 1999-2018 | −1.47 (−2.39 to −0.87) | < 0.001 |
| 2018-2020 | 18.02 (3.71 to 24.50) | < 0.001 |
| **Northeast** | | |
| 1999-2006 | −0.54 (−1.70 to 1.93) | p>0.05 |
| 2006-2009 | −6.97 (−8.73 to −3.05) | < 0.001 |
| 2009-2018 | Not significant |  |
| 2018-2021 | 8.49 (4.29 to 11.00) | < 0.001 |
| 2021-2023 | −7.57 (−12.79 to −1.44) | < 0.001 |
| **Midwest** | | |
| 1999-2018 | −1.24 (−1.79 to −0.81) | < 0.001 |
| 2018-2021 | 15.48 (9.76 to 18.84) | < 0.001 |
| 2021-2023 | −12.33 (−18.81 to −5.03) | < 0.001 |
| **South** | | |
| 1999-2018 | −1.35 (−2.07 to −0.77) | < 0.001 |
| 2018-2021 | 21.51 (14.30 to 25.87) | < 0.001 |
| 2021-2023 | −13.37 (−20.58 to −5.58) | < 0.001 |
| **West** | | |
| 1999-2018 | 0.01 (−0.64 to 0.55) | p>0.05 |
| 2018-2021 | 17.84 (11.75 to 21.34) | < 0.001 |
| 2021-2023 | −12.21 (−18.85 to −5.19) | < 0.001 |

APC = Annual percent change; NH = non-Hispanic. N/A = unreliable or suppressed

**Supplemental Table 4** Overall and Sex‐Stratified Sepsis and Diabetes–related Age-Adjusted Mortality Rates per 100,000 in Adults in the United States from 1999 to 2023

| **Age-Adjusted Rate (95% CI)** | | | |
| --- | --- | --- | --- |
| **Year** | **Men** | **Women** | **Overall** |
| 1999 | 9.24 (9.01–9.46) | 8.64 (8.46–8.82) | 8.88 (8.74–9.01) |
| 2000 | 9.19 (8.97–9.42) | 8.45 (8.27–8.62) | 8.73 (8.59–8.86) |
| 2001 | 9.34 (9.12–9.57) | 8.65 (8.48–8.83) | 8.90 (8.76–9.04) |
| 2002 | 9.53 (9.30–9.75) | 8.71 (8.54–8.89) | 8.99 (8.85–9.13) |
| 2003 | 9.67 (9.45–9.89) | 8.75 (8.57–8.93) | 9.09 (8.95–9.22) |
| 2004 | 9.77 (9.55–9.99) | 8.52 (8.35–8.69) | 9.02 (8.88–9.15) |
| 2005 | 10.15 (9.93–10.37) | 8.81 (8.63–8.98) | 9.35 (9.22–9.49) |
| 2006 | 9.56 (9.35–9.77) | 8.31 (8.14–8.47) | 8.81 (8.68–8.95) |
| 2007 | 9.47 (9.26–9.68) | 7.88 (7.71–8.04) | 8.53 (8.40–8.65) |
| 2008 | 9.11 (8.90–9.31) | 7.68 (7.52–7.84) | 8.27 (8.15–8.40) |
| 2009 | 8.76 (8.56–8.95) | 7.14 (6.99–7.29) | 7.86 (7.74–7.98) |
| 2010 | 8.66 (8.47–8.86) | 7.12 (6.97–7.27) | 7.76 (7.65–7.88) |
| 2011 | 8.51 (8.32–8.70) | 7.14 (6.99–7.30) | 7.72 (7.60–7.84) |
| 2012 | 8.22 (8.04–8.40) | 6.67 (6.52–6.81) | 7.30 (7.19–7.41) |
| 2013 | 8.29 (8.11–8.47) | 6.75 (6.61–6.90) | 7.43 (7.32–7.55) |
| 2014 | 8.42 (8.24–8.60) | 6.55 (6.41–6.69) | 7.35 (7.24–7.46) |
| 2015 | 8.77 (8.59–8.95) | 6.84 (6.70–6.98) | 7.67 (7.56–7.79) |
| 2016 | 9.28 (9.09–9.47) | 6.82 (6.68–6.96) | 7.88 (7.77–8.00) |
| 2017 | 9.25 (9.07–9.43) | 6.77 (6.63–6.91) | 7.82 (7.71–7.93) |
| 2018 | 9.38 (9.20–9.57) | 6.68 (6.55–6.82) | 7.85 (7.74–7.96) |
| 2019 | 9.22 (9.04–9.40) | 6.50 (6.36–6.63) | 7.70 (7.59–7.80) |
| 2020 | 12.96 (12.75–13.16) | 8.88 (8.72–9.03) | 10.68 (10.55–10.81) |
| 2021 | 14.59 (14.37–14.81) | 10.05 (9.88–10.21) | 12.08 (11.94–12.21) |
| 2022 | 12.55 (12.35–12.75) | 8.88 (8.73–9.04) | 10.49 (10.37–10.62) |
| 2023 | 11.25 (11.06–11.44) | 7.83 (7.69–7.97) | 9.33 (9.21–9.45) |
| **Overall** | 9.72 (9.52-9.93) | 7.80 (7.64-7.95) | 8.62 (8.50-8.74) |

**Supplemental Table 5** Sepsis and Diabetes–related Mortality, Stratified by Age group in Adults in the United States, 1999 to 2023.

|  | **Deaths** | | | **Age-Adjusted Rate (95% CI)** | | |
| --- | --- | --- | --- | --- | --- | --- |
| **Year** | **Young Adults** | **Middle Aged Adults** | **Older Adults** | **Young Adults** | **Middle Aged Adults** | **Older Adults** |
| 1999 | 562 | 3322 | 11786 | 0.68 (0.63–0.74) | 5.54 (5.35–5.72) | 34.08 (33.46–34.69) |
| 2000 | 488 | 3411 | 11702 | 0.63 (0.57–0.68) | 5.48 (5.29–5.66) | 33.54 (32.93–34.15) |
| 2001 | 510 | 3702 | 11982 | 0.63 (0.57–0.68) | 5.75 (5.57–5.94) | 33.94 (33.33–34.55) |
| 2002 | 501 | 3845 | 12333 | 0.58 (0.53–0.63) | 5.70 (5.52–5.88) | 34.62 (34.01–35.23) |
| 2003 | 571 | 4054 | 12506 | 0.68 (0.63–0.74) | 5.79 (5.61–5.97) | 34.71 (34.10–35.31) |
| 2004 | 557 | 4061 | 12630 | 0.68 (0.63–0.74) | 5.62 (5.44–5.79) | 34.66 (34.06–35.27) |
| 2005 | 543 | 4333 | 13360 | 0.68 (0.62–0.74) | 5.74 (5.56–5.91) | 36.16 (35.55–36.77) |
| 2006 | 520 | 4342 | 12649 | 0.64 (0.58–0.69) | 5.60 (5.43–5.76) | 33.76 (33.18–34.35) |
| 2007 | 505 | 4232 | 12546 | 0.63 (0.57–0.68) | 5.26 (5.10–5.42) | 32.90 (32.33–33.48) |
| 2008 | 515 | 4112 | 12455 | 0.63 (0.57–0.68) | 5.02 (4.87–5.18) | 32.01 (31.45–32.58) |
| 2009 | 499 | 4069 | 11899 | 0.68 (0.62–0.74) | 4.86 (4.71–5.01) | 30.08 (29.54–30.62) |
| 2010 | 509 | 4100 | 11982 | 0.68 (0.62–0.74) | 4.76 (4.62–4.91) | 29.75 (29.21–30.28) |
| 2011 | 524 | 4278 | 12158 | 0.68 (0.62–0.74) | 4.83 (4.68–4.97) | 29.42 (28.89–29.94) |
| 2012 | 441 | 4110 | 11958 | 0.53 (0.48–0.58) | 4.63 (4.48–4.77) | 27.97 (27.46–28.47) |
| 2013 | 469 | 4183 | 12393 | 0.63 (0.57–0.68) | 4.69 (4.54–4.83) | 28.32 (27.82–28.82) |
| 2014 | 486 | 4269 | 12620 | 0.63 (0.57–0.68) | 4.69 (4.54–4.83) | 27.91 (27.42–28.40) |
| 2015 | 539 | 4435 | 13553 | 0.68 (0.62–0.74) | 4.83 (4.68–4.97) | 29.18 (28.69–29.68) |
| 2016 | 506 | 4684 | 14200 | 0.68 (0.62–0.74) | 5.06 (4.92–5.21) | 29.83 (29.33–30.32) |
| 2017 | 514 | 4665 | 14688 | 0.63 (0.57–0.68) | 4.94 (4.80–5.09) | 29.84 (29.36–30.33) |
| 2018 | 555 | 4658 | 15004 | 0.68 (0.62–0.74) | 5.07 (4.92–5.22) | 29.64 (29.16–30.12) |
| 2019 | 552 | 4657 | 15048 | 0.68 (0.62–0.74) | 5.05 (4.90–5.20) | 28.90 (28.43–29.36) |
| 2020 | 895 | 7044 | 20724 | 1.05 (0.98–1.12) | 7.71 (7.53–7.90) | 38.61 (38.08–39.14) |
| 2021 | 1171 | 8287 | 22557 | 1.42 (1.34–1.50) | 8.99 (8.79–9.18) | 42.67 (42.11–43.23) |
| 2022 | 853 | 6661 | 21231 | 1.00 (0.93–1.07) | 7.33 (7.15–7.51) | 38.44 (37.92–38.96) |
| 2023 | 715 | 5577 | 19652 | 0.84 (0.77–0.90) | 6.10 (5.94–6.26) | 35.05 (34.55–35.54) |
| **Overall** | 14500 | 115091 | 353616 | 0.72 (0.66-0.77) | 5.56 (5.40-5.72) | 32.64 (32.09-33.18) |

Young Adult = 25-44 years; Middle Aged Adults = 45-64 years; Older Adults = 65 years and above

**Supplemental Table 6** Sepsis and Diabetes–related Age-Adjusted Mortality Rates per 100,000 stratified by Race in Adults in the United States from 1999 to 2023

| **Age-Adjusted Rate (95% CI)** | | | | | |
| --- | --- | --- | --- | --- | --- |
| **Year** | **NH White** | **NH Black or African American** | **NH American Indian or Alaska Native** | **Hispanic or Latino** | **NH Asian or Pacific Islander** |
| 1999 | 6.93 (6.80–7.07) | 23.04 (22.27–23.82) | 19.84 (16.55–23.12) | 15.25 (14.41–16.09) | 8.16 (7.23–9.08) |
| 2000 | 6.76 (6.63–6.89) | 23.11 (22.33–23.88) | 16.81 (13.89–19.72) | 15.77 (14.93–16.61) | 7.58 (6.72–8.44) |
| 2001 | 6.92 (6.78–7.05) | 22.82 (22.06–23.58) | 16.25 (13.42–19.08) | 16.47 (15.64–17.30) | 7.59 (6.75–8.43) |
| 2002 | 7.06 (6.92–7.19) | 22.98 (22.22–23.74) | 17.88 (14.96–20.81) | 15.70 (14.92–16.49) | 7.99 (7.18–8.81) |
| 2003 | 7.14 (7.01–7.27) | 22.41 (21.67–23.15) | 16.43 (13.72–19.13) | 15.63 (14.87–16.39) | 8.83 (8.00–9.67) |
| 2004 | 7.14 (7.00–7.27) | 22.21 (21.47–22.94) | 18.10 (15.27–20.92) | 15.34 (14.60–16.07) | 8.02 (7.24–8.80) |
| 2005 | 7.31 (7.18–7.45) | 23.16 (22.43–23.90) | 16.07 (13.49–18.66) | 16.04 (15.30–16.77) | 8.70 (7.92–9.48) |
| 2006 | 6.97 (6.84–7.10) | 20.80 (20.11–21.50) | 16.18 (13.61–18.74) | 14.84 (14.15–15.52) | 8.42 (7.68–9.17) |
| 2007 | 6.71 (6.59–6.84) | 20.67 (19.99–21.36) | 16.38 (13.80–18.96) | 14.77 (14.09–15.44) | 7.63 (6.93–8.32) |
| 2008 | 6.64 (6.52–6.77) | 18.75 (18.11–19.39) | 16.78 (14.16–19.40) | 13.07 (12.46–13.68) | 7.90 (7.21–8.58) |
| 2009 | 6.33 (6.21–6.45) | 16.87 (16.27–17.47) | 16.83 (14.29–19.38) | 12.64 (12.05–13.23) | 7.51 (6.86–8.16) |
| 2010 | 6.25 (6.13–6.37) | 16.63 (16.04–17.22) | 15.40 (13.04–17.75) | 12.71 (12.13–13.29) | 7.09 (6.48–7.71) |
| 2011 | 6.28 (6.16–6.40) | 16.09 (15.52–16.66) | 17.49 (14.98–20.01) | 12.43 (11.88–12.98) | 6.82 (6.24–7.39) |
| 2012 | 6.03 (5.92–6.15) | 14.39 (13.86–14.92) | 17.61 (15.20–20.03) | 11.25 (10.74–11.76) | 6.36 (5.82–6.90) |
| 2013 | 6.11 (6.00–6.23) | 14.10 (13.58–14.61) | 16.21 (13.99–18.43) | 11.65 (11.15–12.16) | 6.65 (6.11–7.19) |
| 2014 | 6.15 (6.04–6.27) | 13.32 (12.83–13.82) | 15.49 (13.34–17.64) | 11.30 (10.82–11.78) | 6.43 (5.92–6.94) |
| 2015 | 6.49 (6.37–6.61) | 13.49 (13.00–13.98) | 16.92 (14.69–19.14) | 11.27 (10.80–11.74) | 6.46 (5.97–6.95) |
| 2016 | 6.70 (6.58–6.82) | 13.91 (13.42–14.40) | 15.82 (13.75–17.88) | 11.51 (11.05–11.97) | 6.07 (5.61–6.54) |
| 2017 | 6.67 (6.55–6.79) | 12.77 (12.31–13.23) | 16.68 (14.59–18.76) | 11.71 (11.26–12.17) | 7.05 (6.57–7.54) |
| 2018 | 6.74 (6.62–6.86) | 13.44 (12.98–13.91) | 16.91 (14.86–18.96) | 10.99 (10.56–11.42) | 6.23 (5.78–6.67) |
| 2019 | 6.60 (6.48–6.71) | 12.79 (12.34–13.24) | 16.06 (14.09–18.03) | 10.75 (10.33–11.16) | 6.38 (5.94–6.82) |
| 2020 | 8.48 (8.35–8.62) | 18.68 (18.15–19.21) | 22.36 (20.12–24.61) | 18.50 (17.97–19.02) | 9.24 (8.73–9.75) |
| 2021 | 10.05 (9.91–10.20) | 19.70 (19.15–20.25) | 28.45 (25.78–31.11) | 18.79 (18.27–19.31) | 10.06 (9.51–10.60) |
| 2022 | 8.97 (8.84–9.11) | 17.28 (16.77–17.79) | 25.07 (22.64–27.51) | 14.87 (14.41–15.34) | 8.33 (7.85–8.81) |
| 2023 | 8.13 (8.00–8.25) | 15.13 (14.66–15.60) | 20.11 (17.95–22.27) | 12.70 (12.28–13.12) | 7.39 (6.95–7.83) |
| **Overall** | 7.02 (6.90–7.15) | 17.94 (17.34-18.54) | 17.92 (15.45-20.40) | 13.84 (13.24–14.43) | 7.55 (6.92-8.18) |

NH=non-Hispanic

**Supplemental Table 7A** Sepsis and Diabetes–related Age-Adjusted Mortality Rates per 100,000 Stratified by State in Adults in the United States, 1999 to 2020.

| **State** | **Age-Adjusted Rate (95% CI)** |
| --- | --- |
| Maine | 4.91 (4.63–5.19) |
| Alaska | 4.93 (4.36–5.49) |
| Florida | 4.97 (4.89–5.04) |
| Idaho | 5.20 (4.90–5.50) |
| New Hampshire | 5.43 (5.11–5.74) |
| Massachusetts | 5.44 (5.30–5.58) |
| Kansas | 5.45 (5.23–5.67) |
| Colorado | 5.48 (5.30–5.66) |
| Arizona | 5.57 (5.42–5.71) |
| North Dakota | 5.61 (5.17–6.05) |
| Nevada | 5.70 (5.46–5.94) |
| Montana | 5.71 (5.35–6.08) |
| Utah | 5.78 (5.50–6.05) |
| Minnesota | 5.92 (5.76–6.09) |
| Oregon | 5.93 (5.74–6.13) |
| Wisconsin | 6.03 (5.87–6.18) |
| Connecticut | 6.03 (5.83–6.23) |
| Missouri | 6.29 (6.14–6.45) |
| Iowa | 6.48 (6.27–6.70) |
| Virginia | 6.62 (6.47–6.77) |
| Nebraska | 6.63 (6.34–6.93) |
| Wyoming | 6.68 (6.12–7.24) |
| Hawaii | 6.80 (6.45–7.14) |
| Delaware | 7.18 (6.74–7.61) |
| Illinois | 7.26 (7.14–7.38) |
| Washington | 7.27 (7.10–7.44) |
| Georgia | 7.31 (7.15–7.46) |
| Pennsylvania | 7.52 (7.41–7.63) |
| Michigan | 7.69 (7.56–7.83) |
| Vermont | 7.88 (7.35–8.42) |
| New York | 7.91 (7.81–8.01) |
| Indiana | 7.95 (7.78–8.13) |
| New Mexico | 8.21 (7.89–8.53) |
| South Dakota | 8.25 (7.77–8.74) |
| Alabama | 8.76 (8.54–8.97) |
| Louisiana | 8.80 (8.57–9.03) |
| Arkansas | 8.97 (8.70–9.24) |
| Rhode Island | 9.18 (8.74–9.62) |
| Ohio | 9.34 (9.21–9.48) |
| Tennessee | 9.38 (9.19–9.58) |
| North Carolina | 9.55 (9.39–9.71) |
| New Jersey | 9.74 (9.58–9.90) |
| South Carolina | 10.56 (10.32–10.79) |
| Maryland | 10.71 (10.49–10.93) |
| California | 11.09 (11.00–11.18) |
| West Virginia | 11.25 (10.89–11.61) |
| Mississippi | 11.50 (11.18–11.82) |
| Texas | 12.20 (12.08–12.33) |
| Kentucky | 12.34 (12.07–12.60) |
| Oklahoma | 14.07 (13.76–14.38) |
| District of Columbia | 18.78 (17.85–19.71) |
| **Overall** | 7.93 (7.66–8.19) |

**Supplemental Table 7B** Sepsis and Diabetes–related Age-Adjusted Mortality Rates per 100,000 Stratified by State in Adults in the United States, 2021 to 2023.

| **State** | **Age-Adjusted Rate (95% CI)** |
| --- | --- |
| Maine | 2.08 (1.64–2.60) |
| Connecticut | 4.10 (3.68–4.51) |
| Massachusetts | 5.42 (5.07–5.77) |
| Hawaii | 5.61 (4.83–6.39) |
| Alaska | 6.07 (4.79–7.59) |
| New Hampshire | 6.22 (5.41–7.02) |
| Illinois | 6.28 (5.99–6.56) |
| Missouri | 7.20 (6.77–7.63) |
| Arizona | 7.40 (7.00–7.80) |
| Michigan | 7.57 (7.23–7.91) |
| Virginia | 7.70 (7.32–8.08) |
| New Jersey | 7.76 (7.40–8.12) |
| New York | 7.79 (7.55–8.04) |
| Florida | 8.18 (7.95–8.40) |
| North Dakota | 8.25 (6.87–9.63) |
| Rhode Island | 8.37 (7.29–9.45) |
| Oregon | 8.41 (7.86–8.96) |
| Kansas | 8.49 (7.80–9.19) |
| Pennsylvania | 8.61 (8.30–8.92) |
| Ohio | 8.61 (8.27–8.95) |
| Alabama | 9.03 (8.50–9.57) |
| North Carolina | 9.32 (8.94–9.70) |
| Wisconsin | 9.37 (8.87–9.86) |
| Utah | 9.53 (8.73–10.33) |
| Vermont | 9.54 (8.10–10.99) |
| Montana | 9.77 (8.62–10.93) |
| Iowa | 10.06 (9.35–10.76) |
| Colorado | 10.18 (9.62–10.75) |
| Georgia | 10.21 (9.79–10.62) |
| Idaho | 10.23 (9.28–11.19) |
| Washington | 10.60 (10.12–11.08) |
| Wyoming | 10.92 (9.16–12.68) |
| Minnesota | 10.97 (10.42–11.52) |
| Louisiana | 11.04 (10.41–11.68) |
| Nevada | 11.31 (10.54–12.09) |
| Delaware | 11.48 (10.19–12.76) |
| Nebraska | 11.57 (10.59–12.56) |
| Indiana | 11.70 (11.16–12.23) |
| Tennessee | 12.31 (11.77–12.84) |
| Arkansas | 12.42 (11.60–13.23) |
| New Mexico | 12.75 (11.75–13.74) |
| West Virginia | 13.43 (12.37–14.48) |
| Maryland | 13.83 (13.22–14.43) |
| Texas | 14.41 (14.10–14.72) |
| California | 15.25 (14.99–15.51) |
| South Dakota | 15.92 (14.17–17.67) |
| District of Columbia | 17.43 (15.15–19.71) |
| South Carolina | 17.63 (16.90–18.36) |
| Mississippi | 21.93 (20.82–23.05) |
| Kentucky | 22.49 (21.58–23.39) |
| Oklahoma | 28.27 (27.17–29.37) |
| **Overall** | 10.69 (9.95–11.42) |

**Supplemental Table 8.** Sepsis and Diabetes–related Age-Adjusted Mortality Rate per 100,000 Stratified by Census Region in Adults in the United States 1999-2023.

| **Census Region** | **Year** | **Age-Adjusted Rate (95% CI)** |
| --- | --- | --- |
| Northeast | 1999 | 9.10 (8.79–9.40) |
| Northeast | 2000 | 8.99 (8.68–9.29) |
| Northeast | 2001 | 9.43 (9.12–9.74) |
| Northeast | 2002 | 8.98 (8.68–9.28) |
| Northeast | 2003 | 8.73 (8.43–9.02) |
| Northeast | 2004 | 8.80 (8.50–9.09) |
| Northeast | 2005 | 9.20 (8.90–9.50) |
| Northeast | 2006 | 8.63 (8.34–8.92) |
| Northeast | 2007 | 8.24 (7.96–8.52) |
| Northeast | 2008 | 7.61 (7.35–7.88) |
| Northeast | 2009 | 6.85 (6.59–7.10) |
| Northeast | 2010 | 6.99 (6.74–7.25) |
| Northeast | 2011 | 7.04 (6.79–7.30) |
| Northeast | 2012 | 6.60 (6.36–6.84) |
| Northeast | 2013 | 6.81 (6.57–7.06) |
| Northeast | 2014 | 6.74 (6.50–6.98) |
| Northeast | 2015 | 6.74 (6.50–6.98) |
| Northeast | 2016 | 6.52 (6.29–6.76) |
| Northeast | 2017 | 6.27 (6.04–6.50) |
| Northeast | 2018 | 6.14 (5.92–6.37) |
| Northeast | 2019 | 6.08 (5.86–6.31) |
| Northeast | 2020 | 7.78 (7.53–8.03) |
| Northeast | 2021 | 7.76 (7.51–8.01) |
| Northeast | 2022 | 7.43 (7.19–7.67) |
| Northeast | 2023 | 6.71 (6.48–6.94) |
| Northeast | **Overall** | 7.61 (7.34–7.87) |
| Midwest | 1999 | 7.92 (7.65–8.19) |
| Midwest | 2000 | 7.72 (7.46–7.99) |
| Midwest | 2001 | 7.63 (7.37–7.89) |
| Midwest | 2002 | 7.72 (7.46–7.98) |
| Midwest | 2003 | 8.17 (7.9–8.44) |
| Midwest | 2004 | 8.08 (7.82–8.35) |
| Midwest | 2005 | 8.29 (8.02–8.55) |
| Midwest | 2006 | 7.77 (7.51–8.03) |
| Midwest | 2007 | 7.73 (7.48–7.99) |
| Midwest | 2008 | 7.39 (7.14–7.63) |
| Midwest | 2009 | 7.19 (6.95–7.44) |
| Midwest | 2010 | 6.79 (6.55–7.02) |
| Midwest | 2011 | 7.06 (6.82–7.29) |
| Midwest | 2012 | 6.49 (6.26–6.71) |
| Midwest | 2013 | 6.56 (6.33–6.78) |
| Midwest | 2014 | 6.64 (6.41–6.86) |
| Midwest | 2015 | 6.8 (6.57–7.03) |
| Midwest | 2016 | 6.65 (6.43–6.88) |
| Midwest | 2017 | 6.65 (6.43–6.87) |
| Midwest | 2018 | 6.8 (6.58–7.02) |
| Midwest | 2019 | 6.7 (6.48–6.92) |
| Midwest | 2020 | 9.21 (8.96–9.46) |
| Midwest | 2021 | 9.8 (9.53–10.07) |
| Midwest | 2022 | 8.66 (8.41–8.9) |
| Midwest | 2023 | 7.68 (7.45–7.91) |
| Midwest | **Overall** | 7.52 (7.28–7.77) |
| South | 1999 | 9.66 (9.41–9.9) |
| South | 2000 | 9.9 (9.66–10.14) |
| South | 2001 | 10.2 (9.96–10.45) |
| South | 2002 | 10.14 (9.9–10.38) |
| South | 2003 | 9.94 (9.71–10.18) |
| South | 2004 | 10.23 (9.99–10.47) |
| South | 2005 | 9.53 (9.3–9.75) |
| South | 2006 | 9.13 (8.91–9.35) |
| South | 2007 | 9 (8.79–9.22) |
| South | 2008 | 8.52 (8.32–8.73) |
| South | 2009 | 8.35 (8.15–8.56) |
| South | 2010 | 7.98 (7.78–8.17) |
| South | 2011 | 7.8 (7.6–7.99) |
| South | 2012 | 7.84 (7.65–8.03) |
| South | 2013 | 7.75 (7.56–7.93) |
| South | 2014 | 8.16 (7.97–8.35) |
| South | 2015 | 8.65 (8.45–8.84) |
| South | 2016 | 8.57 (8.38–8.76) |
| South | 2017 | 8.81 (8.62–9) |
| South | 2018 | 8.56 (8.37–8.74) |
| South | 2019 | 12.13 (11.91–12.34) |
| South | 2020 | 14.25 (14.01–14.49) |
| South | 2021 | 11.99 (11.77–12.2) |
| South | 2022 | 10.72 (10.52–10.92) |
| South | 2023 | 9.75 (9.5–9.99) |
| South | **Overall** | 9.50 (9.29–9.72) |
| West | 1999 | 8.14 (7.85–8.44) |
| West | 2000 | 7.84 (7.55–8.13) |
| West | 2001 | 7.98 (7.7–8.27) |
| West | 2002 | 8.44 (8.15–8.73) |
| West | 2003 | 8.66 (8.36–8.95) |
| West | 2004 | 8.65 (8.36–8.94) |
| West | 2005 | 9.13 (8.83–9.42) |
| West | 2006 | 8.83 (8.54–9.11) |
| West | 2007 | 8.63 (8.35–8.91) |
| West | 2008 | 8.53 (8.25–8.81) |
| West | 2009 | 8.12 (7.85–8.38) |
| West | 2010 | 8.41 (8.14–8.67) |
| West | 2011 | 8.53 (8.26–8.79) |
| West | 2012 | 7.95 (7.7–8.2) |
| West | 2013 | 8.04 (7.79–8.3) |
| West | 2014 | 7.91 (7.67–8.16) |
| West | 2015 | 8.45 (8.2–8.7) |
| West | 2016 | 8.8 (8.55–9.06) |
| West | 2017 | 9.07 (8.82–9.33) |
| West | 2018 | 8.67 (8.42–8.91) |
| West | 2019 | 8.47 (8.23–8.71) |
| West | 2020 | 11.96 (11.68–12.24) |
| West | 2021 | 14.07 (13.76–14.37) |
| West | 2022 | 12.03 (11.75–12.3) |
| West | 2023 | 10.64 (10.38–10.9) |
| West | **Overall** | 9.04 (8.77–9.31) |

**Supplemental Table 9.** Overall Sepsis and Diabetes–related Age-Adjusted Mortality Rates per 100,000 in Adults in the Metropolitan and Non-metropolitan areas in the United States, 1999 to 2020.

| **Age-Adjusted Rate (95% CI)** | | |
| --- | --- | --- |
| **Year** | **Metropolitan** | **Nonmetropolitan** |
| 1999 | 8.98 (8.83–9.14) | 8.36 (8.04–8.67) |
| 2000 | 8.88 (8.73–9.03) | 7.95 (7.65–8.25) |
| 2001 | 9.01 (8.86–9.17) | 8.42 (8.11–8.73) |
| 2002 | 9.08 (8.93–9.23) | 8.67 (8.36–8.98) |
| 2003 | 9.09 (8.94–9.24) | 9.08 (8.76–9.4) |
| 2004 | 9.03 (8.88–9.17) | 9.02 (8.7–9.34) |
| 2005 | 9.48 (9.33–9.64) | 8.9 (8.59–9.21) |
| 2006 | 8.91 (8.76–9.05) | 8.37 (8.06–8.67) |
| 2007 | 8.57 (8.43–8.72) | 8.39 (8.09–8.69) |
| 2008 | 8.25 (8.11–8.39) | 8.47 (8.17–8.77) |
| 2009 | 7.74 (7.60–7.87) | 8.18 (7.88–8.47) |
| 2010 | 7.69 (7.56–7.82) | 8.08 (7.79–8.37) |
| 2011 | 7.64 (7.51–7.77) | 8.11 (7.82–8.4) |
| 2012 | 7.21 (7.08–7.33) | 7.86 (7.58–8.14) |
| 2013 | 7.27 (7.15–7.39) | 8.15 (7.86–8.44) |
| 2014 | 7.20 (7.08–7.32) | 8.24 (7.95–8.53) |
| 2015 | 7.49 (7.37–7.61) | 8.65 (8.35–8.94) |
| 2016 | 7.61 (7.49–7.73) | 9.22 (8.92–9.52) |
| 2017 | 7.59 (7.47–7.71) | 9.19 (8.89–9.49) |
| 2018 | 7.56 (7.44–7.68) | 9.51 (9.21–9.81) |
| 2019 | 7.31 (7.20–7.43) | 9.68 (9.38–9.98) |
| 2020 | 10.35 (10.21–10.48) | 12.64 (12.29–12.99) |
| **Overall** | 8.27 (8.13–8.41) | 8.78 (8.48–9.08) |

**Supplementary Figure 1: Diabetes Mellitus and Sepsis-Related Annual Percentage Change (APC) in the United States from 1999 to 2023 stratified by (A) Overall (B) Sex (C) Race (D) Urbanization (E) Consensus Region (F) Age**

1. **Overall**


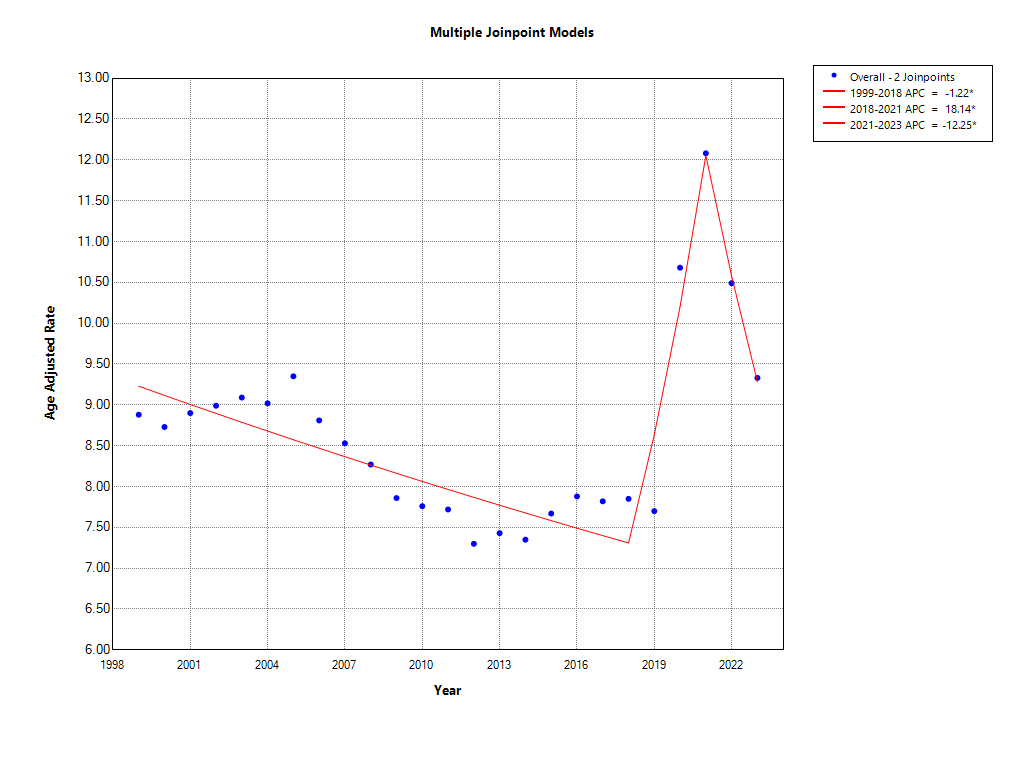


1. **Sex**


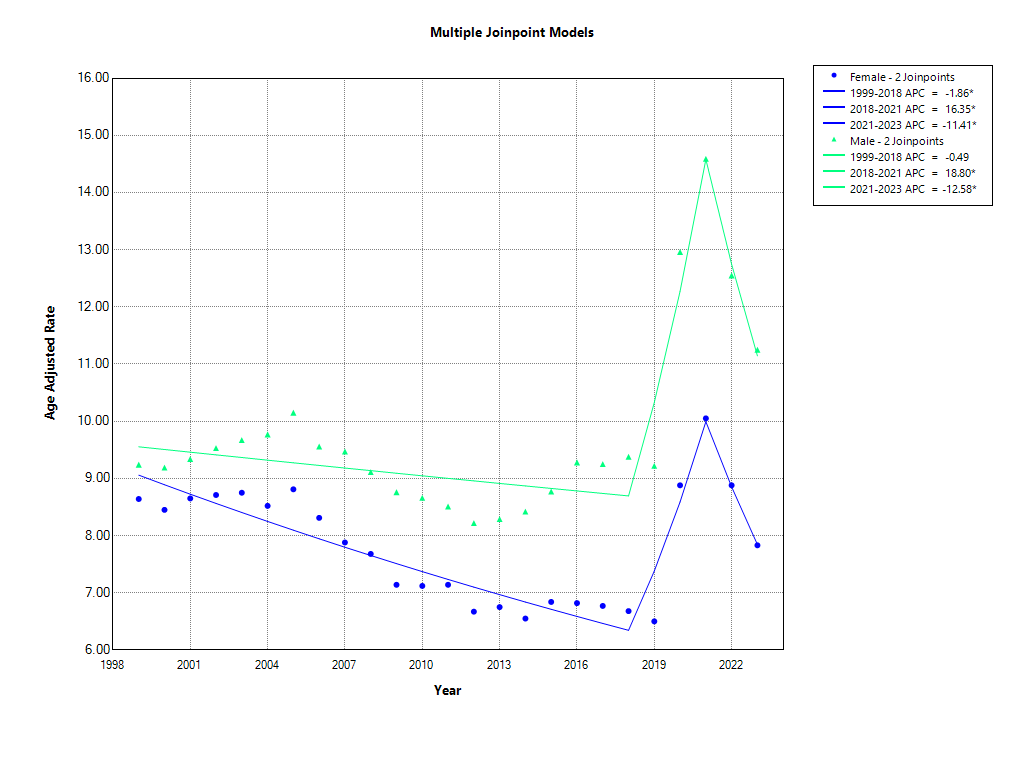


1. **Race**


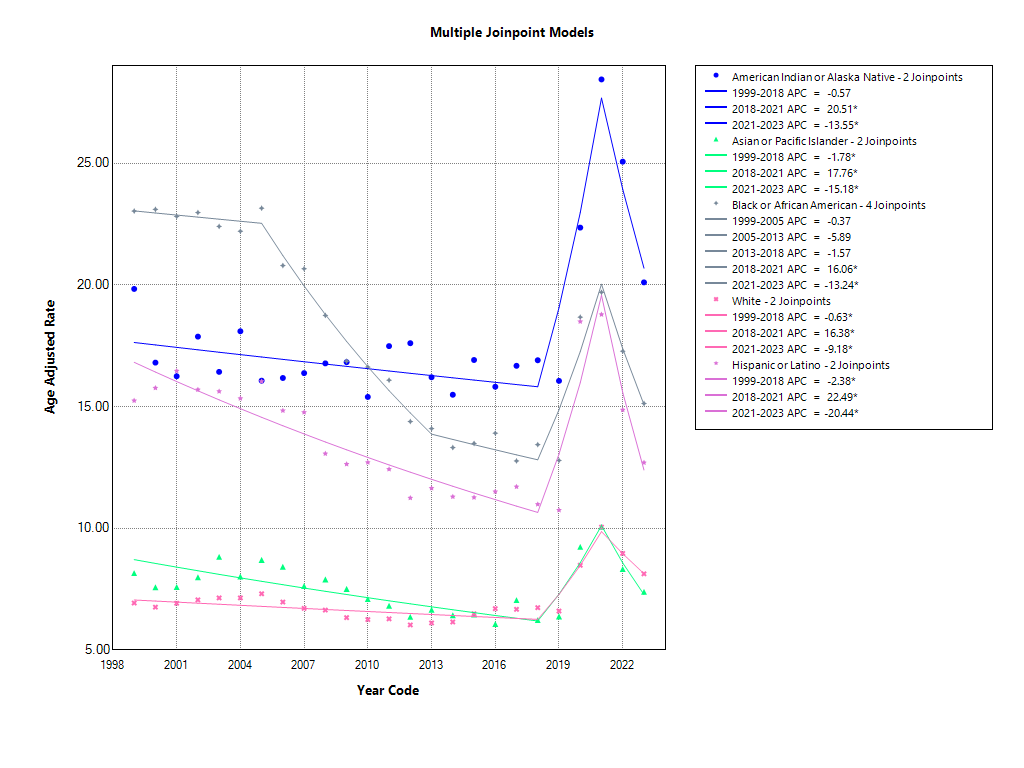


1. **Urbanization**


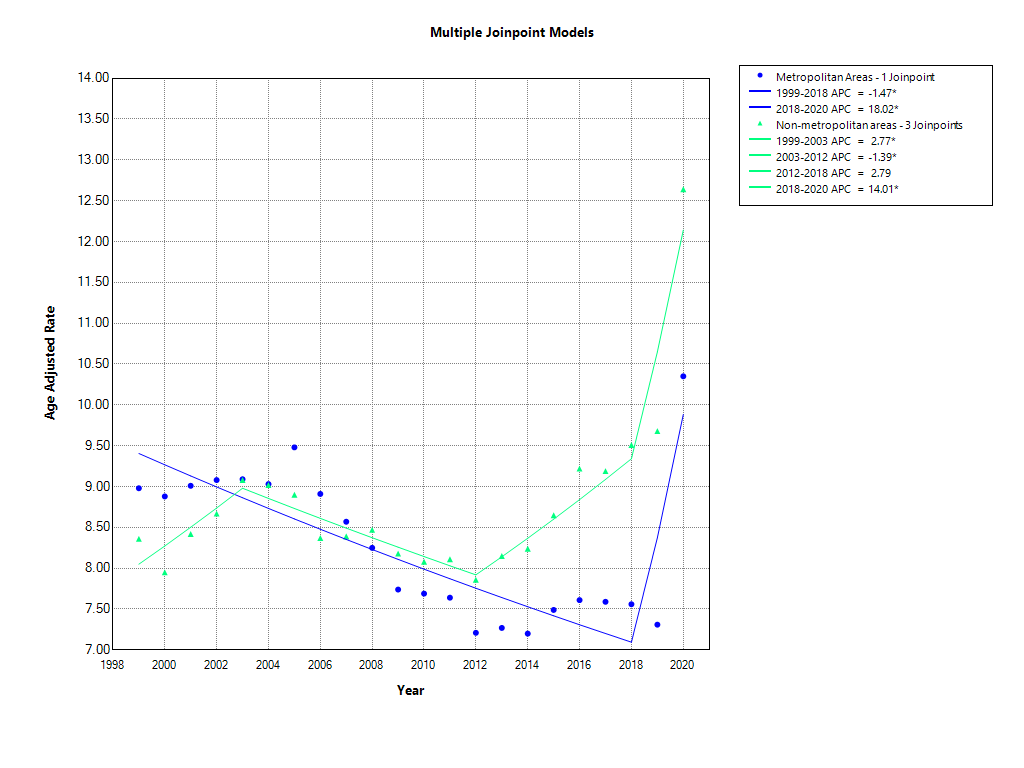


**E) Consensus Region**


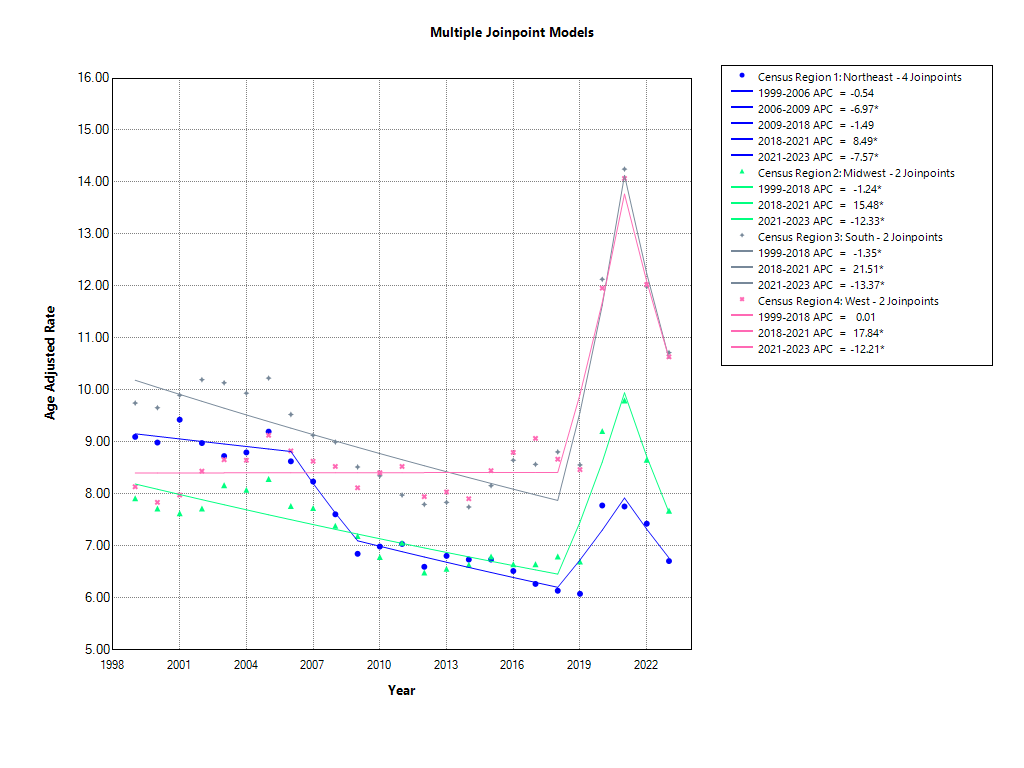


**F) Age**


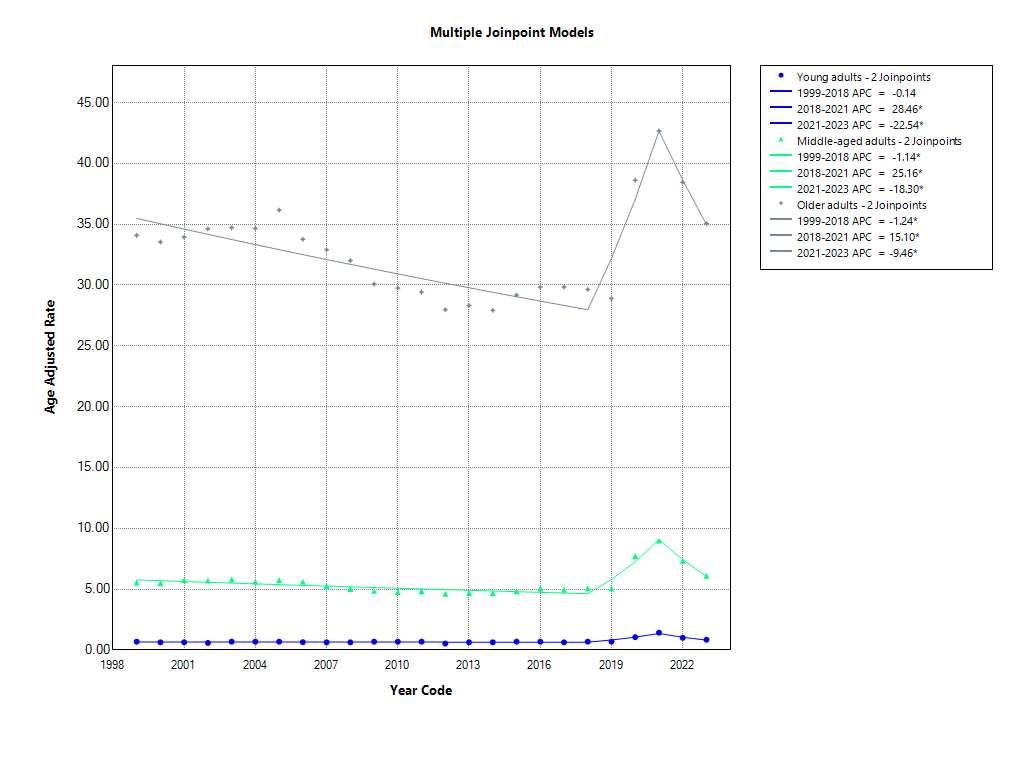


**Supplementary Figure 2: State-wise distribution of sepsis related mortality in diabetic patients (A) 1999-2020 (B) 2021-2023**

**
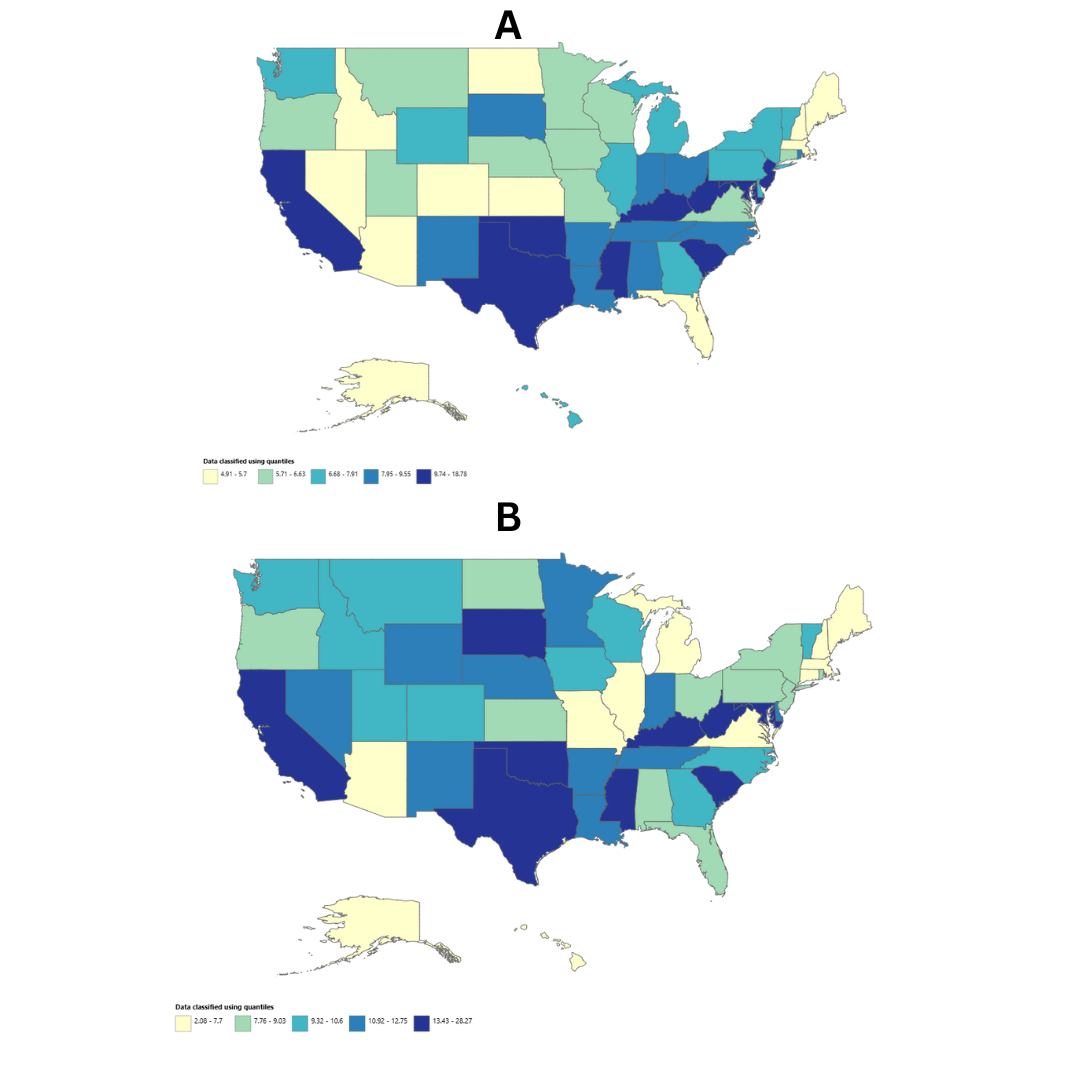
**
